# Integrative single-cell and bulk transcriptomic analysis identifies monocyte/macrophage senescence-associated candidate genes in pulmonary arterial hypertension

**DOI:** 10.3389/fimmu.2026.1938985

**Published:** 2026-10-12

**Authors:** Xiaojun Chen, Weibin Wu, Yilong Liu, Guili Lian, Qingquan Liu

**Affiliations:** 1 Department of Cardiology, The Second Affiliated Hospital of Fujian Medical University, Quanzhou, Fujian, China; 2 Department of Cardiology, The First Affiliated Hospital of Xiamen University, School of Medicine, Xiamen University, Xiamen, Fujian, China; 3 Department of Geriatrics, The First Affiliated Hospital of Fujian Medical University, Fuzhou, Fujian, China; 4 Fujian Hypertension Research Institute, The First Affiliated Hospital of Fujian Medical University, Fuzhou, Fujian, China

**Keywords:** cellular senescence, monocytes/macrophages, pulmonary arterial hypertension, senescence-associated candidate genes, single-cell RNA sequencing

## Abstract

**Background:**

Pulmonary arterial hypertension (PAH) is a progressive vascular disorder characterized by immune dysregulation and vascular remodeling. Cellular senescence modulates immune responses, but its role in PAH, particularly in monocytes/macrophages, remains unclear. Here, we integrated single-cell and bulk transcriptomic data to identify senescence-associated candidate genes associated with monocytes/macrophages in PAH.

**Methods:**

Publicly available single-cell RNA-seq data from PAH and control pulmonary vascular tissues were analyzed. A senescence-associated score was calculated for each cell based on MSigDB gene sets. Senescence-associated genes in monocytes/macrophages were identified through correlation and differential expression screening, and further examined in bulk transcriptomic datasets. Bulk transcriptomic discovery used GSE113439, GSE117261, and GSE254617. A sample-title audit identified 68 shared PHBI identifiers between GSE117261 and GSE254617; therefore, the merged data represented 257 expression profiles from 189 unique subjects. LASSO, Random Forest, and XGBoost were used as complementary feature-selection approaches, and genes jointly retained by the three algorithms were defined as candidates for evaluation in independent cohorts.

**Results:**

Single-cell RNA-seq identified 14 cell types in PAH pulmonary vascular tissues, with dendritic cells, T cells, and monocytes/macrophages exhibiting the highest senescence-associated scores. Integrative screening identified 405 senescence-associated genes enriched in monocytes/macrophages. Multi-algorithm screening prioritized 10 candidate genes for subsequent evaluation. After within-cohort multiple-testing correction, external cohort analyses reproduced the discovery-stage expression directions for *RGS1*, *PHLDA1*, *MAFB*, and *RNF13* in lung tissue cohorts, and for MAFB in a PBMC cohort. With nominal *P* values, *RGS1* was significantly upregulated in GSE48149 and GSE53408 (both lung), and *PHLDA1* was upregulated in GSE48149 (lung). *MAFB* was significantly downregulated in GSE33463 (PBMC) and GSE48149 (lung), whereas *RNF13* was downregulated in GSE15197 and GSE48149 (both lung). Functional analysis revealed significant enrichment of immune-inflammatory and phagocytosis-related pathways among the candidate genes. Consensus clustering based on the candidate genes classified PAH patients into two exploratory transcriptomic groups with distinct expression and pathway activity profiles. *In vivo* evaluation in an MCT-induced PAH rat model showed seven significant direction-consistent changes, two direction-consistent nonsignificant trends, and one discordant gene (*PDE4B*), supporting tissue-level expression concordance.

**Conclusion:**

These analyses identify a Mono.Macs-associated senescence-related candidate gene set in PAH. Independent expression analyses provide additional support for *RGS1*, *PHLDA1*, *MAFB*, and *RNF13* as candidate genes for subsequent mechanistic and functional validation.

## 1 Introduction

Pulmonary arterial hypertension (PAH) is a progressive pulmonary vascular disease characterized by persistent elevation of pulmonary arterial pressure due to increased pulmonary vascular resistance, ultimately leading to right ventricular failure and premature death (1). Pulmonary vascular remodeling, coupled with chronic inflammation and immune dysregulation, is a key pathological feature of PAH (2, 3). Although multiple vascular cell types, including endothelial cells, smooth muscle cells, and fibroblasts, contribute to disease progression, accumulating evidence indicates that immune cells play critical roles in shaping the pulmonary vascular microenvironment (4, 5). However, how senescence-associated changes in immune cells are linked to PAH progression remains unclear.

Cellular senescence is characterized by stable cell-cycle arrest, metabolic alterations, and secretion of pro-inflammatory mediators collectively termed the senescence-associated secretory phenotype (SASP) (6, 7). Recent studies have implicated cellular senescence in pulmonary vascular remodeling and inflammatory microenvironment alterations in PAH (8, 9). Cellular senescence can contribute to pulmonary vascular remodeling by promoting endothelial dysfunction, pro-inflammatory signaling, and extracellular matrix remodeling through SASP-mediated mechanisms (10). In addition, immune cells have increasingly been recognized as important contributors to pulmonary vascular inflammation and remodeling, accompanied by profound immune dysregulation (2, 3, 11).

Previous studies have demonstrated that monocytes/macrophages accumulate around pulmonary vessels and promote chemokine production, extracellular matrix remodeling, and pro-remodeling signaling within the pulmonary vascular microenvironment (12). In addition, monocytes/macrophages with senescence-associated features have been associated with metabolic reprogramming, impaired phagocytic function, and persistent pro-inflammatory signaling, potentially exacerbating immune dysregulation and tissue remodeling (13). Nevertheless, the molecular characteristics of monocytes/macrophages with senescence-associated features in PAH remain unclear.

Single-cell RNA sequencing (scRNA-seq) enables high-resolution dissection of cellular heterogeneity and cell-type-specific transcriptional programs in complex diseases. Unlike bulk RNA-seq, which measures averaged gene expression across mixed cell populations, scRNA-seq facilitates the identification of rare cellular states and disease-associated transcriptional alterations at single-cell resolution (14, 15). Recent scRNA-seq studies in PAH have identified diverse immune and vascular cell subsets, as well as transcriptional programs associated with pulmonary vascular remodeling (16, 17). However, systematic analyses of senescence-associated alterations in monocytes/macrophages and the prioritization of candidate genes in PAH are still limited.

In the present study, we integrated scRNA-seq and bulk transcriptomic datasets to characterize senescence-associated transcriptional heterogeneity across cell types in PAH pulmonary vascular tissues. We also examined the relationships between the senescence-associated transcriptional signature and cellular stemness, metabolic activity, and intercellular communication within the pulmonary vascular context. Given the important role of monocytes/macrophages in the PAH microenvironment, we subsequently prioritized Mono.Macs-associated candidate genes through integrative machine learning-based multi-algorithm screening. The expression of these prioritized candidate genes was further evaluated at the tissue level in an MCT-induced PAH rat model.

## 2 Methods

### 2.1 Single-cell RNA-seq data processing

Single-cell RNA-seq dataset GSE210248 was obtained from GEO, derived from dissected pulmonary artery/perivascular tissue, comprising 3 healthy donors and 3 PAH patients, with cell annotations provided by the original authors (18). Data were processed using Seurat (version 4.0.0) (19). After quality control (excluding cells with < 200 or > 6,000 detected genes and > 20% mitochondrial counts), 22,704 cells were retained. Data were normalized using LogNormalize (scale factor = 10,000), and highly variable genes were identified using FindVariableFeatures. Principal component analysis (PCA) was performed, and the top 30 principal components were selected. Batch effects were corrected using Harmony (version 1.0.0), and UMAP was performed based on the Harmony-corrected embeddings (20). Cell clusters were identified using the Louvain algorithm. Multiple resolutions ranging from 0.01 to 1.0 were tested, and the optimal resolution was selected using clustree (21). Marker genes for each cluster were identified using the Wilcoxon rank-sum test via FindAllMarkers (|log_2_FC| > 0.25, adjusted *P* < 0.05). Cell cycle scores were calculated using CellCycleScoring based on canonical G1/S and G2/M gene sets. Cell-type-specific marker genes were identified using the COSG algorithm (mu = 1, n_genes_user = 100) (22). The top 100 marker genes per cell type were selected; the top 3 were visualized in a heatmap, and the top 100 were subjected to KEGG enrichment analysis using clusterProfiler (version 4.4.4), retaining the top 3 enriched pathways (23). All human data analyzed in this study were obtained from publicly available GEO datasets; no additional ethical approval was required for secondary analysis of these de-identified data.

### 2.2 Senescence scoring and differential abundance analysis

A senescence-associated score was calculated for each cell using AddModuleScore (seed = 42), based on 321 senescence-related genes from MSigDB (REACTOME_CELLULAR_SENESCENCE, GOBP_CELLULAR_SENESCENCE, and KAMMINGA_SENESCENCE) (24). For each gene, 100 control genes were randomly selected from the same expression bin. Cells were stratified into high- and low-senescence-associated score groups based on the median score. The median split was chosen for descriptive stratification to generate balanced comparison groups for visualization, not as a biological threshold. To assess the robustness of the senescence-associated score, an independent UCell rank-based scoring approach was applied using the same 321-gene set. Spearman correlation between AddModuleScore and UCell scores was calculated within Mono.Macs. Donor-stratified associations between each candidate gene and UCell score were assessed using Spearman correlation. Overlap between the 321 senescence genes and the 10 candidate genes was audited, and pathway-level overlap was assessed. In addition to the median split for visualization, continuous senescence-associated scores were used for correlation analyses within Mono.Macs.

Senescence-associated scores were visualized using box plots and three-panel UMAP plots, showing cell type distribution, score distribution, and group assignment. Differential abundance between groups was assessed using three methods. First, cell counts and proportions were compared using bar plots. Second, transcriptomic neighborhood-based differential abundance was analyzed using miloR (version 1.4.0) with k = 5, d = 30, and prop = 0.2; results were visualized using beeswarm plots (25). Third, odds ratios (ORs) were calculated using Fisher’s exact test with 95% confidence intervals, and results were visualized using bubble plots. In these analyses, the inferential unit was the cell-level neighborhood or cell-type category, not the donor. Composition and cell-number sensitivity analyses were not performed for these differential abundance analyses; therefore, differential abundance results should be interpreted as descriptive and exploratory.

### 2.3 Stemness, pathway activity, and cell communication

Cellular stemness was assessed using the CytoTRACE algorithm (26). The distribution of CytoTRACE scores across all cells in the transcriptional embedding was visualized by UMAP. CytoTRACE scores, ranging from 0 to 1 with higher scores indicating greater stemness potential, were visualized using violin plots to compare distributions between high- and low-senescence-associated score groups. Mean scores for each cell type were visualized in a heatmap. Pathway activity scores for Hallmark and metabolic pathways were calculated using the AddModuleScore function, and differences between high- and low-senescence-associated score groups were evaluated and visualized using waterfall plots; metabolic pathway scores based on KEGG and Reactome gene sets were calculated similarly.

Cell-cell communication was analyzed using CellChat (version 1.6.0) with CellChatDB.human (27). These results represent inferred communication associated with the senescence-associated score. Communication networks were constructed separately for the high- and low-senescence-associated score groups. Interaction numbers and strengths between cell types were visualized using network and bar plots. Ligand-receptor interactions were identified using identifyOverExpressedGenes and identifyOverExpressedInteractions, communication probabilities were computed with computeCommunProb (min.cells = 10), and pathway-level communication was summarized with computeCommunProbPathway. The two CellChat objects were merged for comparison. Altered signaling pathways were identified using dot plots, ligand-receptor pairs enhanced or weakened in the low group were visualized using bubble plots, and the top 20 ligand-receptor pairs and pathway interaction differences were displayed in bar plots. Composition and cell-number sensitivity analyses were not performed for CellChat inference; therefore, these inferred communication patterns should be interpreted as descriptive and exploratory.

### 2.4 Screening of Mono.Macs-enriched senescence-related genes

A two-step screening strategy was employed to identify genes associated with senescence and enriched in Mono.Macs. First, within the Mono.Macs population, Spearman correlation analysis was performed to identify genes positively correlated with the senescence score (ρ > 0, adjusted *P* < 0.05). Second, differential expression analysis between Mono.Macs and other cell types was performed using the FindMarkers function with thresholds of log_2_FC > 0.1 and adjusted *P* < 0.05. Ribosomal protein genes (with prefix RP[SL]) were excluded from the analysis. The results of the Spearman correlation analysis were visualized as a volcano plot, showing the correlation coefficients and adjusted P-values. The top 20 candidate genes were visualized using a bubble plot, and a bar plot was used to compare their mean expression levels between Mono.Macs and other cell types.

### 2.5 Bulk transcriptomic data processing

Bulk transcriptomic datasets GSE113439 (microarray; 15 PAH, 11 healthy controls), GSE117261 (microarray; 58 PAH, 25 healthy controls), and GSE254617 (RNA-seq; 96 PAH, 52 healthy controls) were downloaded from GEO (28–30). Microarray datasets (GSE113439 and GSE117261) were checked for expression distribution, log_2_-transformed when necessary, and collapsed to gene level by selecting the probe with the largest interquartile range across all samples. Expression values were then quantile-normalized. The RNA-seq dataset (GSE254617) was aligned to GENCODE v32 using HISAT2, quantified with StringTie, and summarized to transcript-level counts with tximport. For the sensitivity analysis described below, GSE254617 counts were additionally filtered for low expression, TMM-normalized, and log_2_-CPM transformed before being combined with the microarray data. Before ComBat, all datasets were quantile-normalized to place expression values on a comparable scale. Batch correction was then performed using the ComBat function in the sva package (version 3.44.0) (31).

The three datasets were merged, yielding a combined dataset of 18,685 genes across 257 expression profiles. Cross-dataset auditing of GEO sample titles identified 68 shared PHBI identifiers between GSE117261 and GSE254617, indicating cross-platform profiles from the same individuals; thus, these datasets corresponded to 189 unique subjects rather than 257 independent individuals. The merged dataset (257 expression profiles) was used for discovery-stage analyses, with this duplication transparently noted. To address the non-independence of repeated PHBI subjects, a sensitivity analysis restricted to 189 unique subjects was performed. One expression profile per individual was retained, preferentially retaining GSE254617 profiles. In the primary deduplicated branch, the original preprocessing pipeline was retained without additional TMM normalization. In the main sensitivity branch, GSE254617 counts were additionally TMM-normalized and log2-CPM transformed before integration. In a secondary prespecified sensitivity analysis, profiles favoring retention of GSE117261 were also evaluated. The 10 prioritized candidate genes were re-evaluated in this unique-subject dataset. To assess the robustness of downstream analyses to the non-independence of repeated PHBI subjects, sensitivity analyses restricted to 189 unique subjects were also performed for GSEA, ssGSEA, and consensus clustering.

The distribution of the three datasets before and after batch correction was visualized using 3D PCA plots. Differentially expressed genes (DEGs) between PAH patients and healthy controls were identified using the limma package (version 3.52.2) with thresholds of |log_2_FC| > 0.5 and adjusted *P* < 0.05 *(*32). Gene Ontology (GO) enrichment analyses, including Biological Process (BP), Cellular Component (CC), and Molecular Function (MF), were performed using the clusterProfiler package. KEGG enrichment analysis was also performed using the same package.

### 2.6 Machine learning-based candidate gene prioritization

To refine the candidate list, three machine learning algorithms were applied as a multi-algorithm candidate prioritization strategy rather than for clinical prediction modeling. LASSO regression was performed using glmnet (version 4.1.4) with alpha = 1 and 10-fold cross-validation; the optimal lambda value (lambda.min) was selected (33). Random Forest analysis was performed using randomForest (version 4.7.1) with 500 trees, and variable importance was evaluated based on the mean decrease in accuracy. XGBoost was performed using xgboost (version 1.7.1) with max_depth = 6, eta = 0.5, objective = ‘binary:logistic’, and nround = 25. The top 15 genes from Random Forest and XGBoost were selected, and the intersection of genes identified by the three algorithms was defined as prioritized candidate genes. Correlations among these candidate genes were visualized using a chord diagram, and discriminatory capacity was evaluated using ROC curves generated with pROC (version 1.18.0) within the discovery-stage dataset; these results are presented as discovery-stage observations rather than validated clinical performance (34). In the bulk dataset, Spearman correlation coefficients were calculated between each candidate gene and all other genes. Positively correlated genes were selected, and their expression patterns were visualized using heatmaps. For all resampling procedures, including LASSO cross-validation, profiles from the same individual were not allowed to contribute independently across folds.

### 2.7 External validation and expression replication

The expression of candidate genes was evaluated in independent PAH-versus-control transcriptomic cohorts, including lung tissue cohorts (GSE15197, GSE48149, GSE53408) and PBMC/whole-blood cohorts (GSE33463, GSE22356, GSE131793, GSE38267). Candidate genes and their expected expression directions were prespecified before external testing, and the external datasets were not used for feature selection. When multiple probes mapped to the same gene, the probe with the largest interquartile range across all samples was selected without using group labels. Two-sided Welch’s t tests were used for targeted PAH-control comparisons, and nominal *P* values were reported. Mean differences were calculated as PAH minus control. Effect sizes were calculated as Hedges’g. Within each cohort, *P* values were adjusted for multiple testing using the Benjamini-Hochberg method across all testable candidate genes.

### 2.8 Single-cell expression visualization and immune infiltration analysis

Expression patterns of the candidate genes were visualized in the discovery-stage single-cell data using the DotPlot and FeaturePlot functions in Seurat, visualizing average expression levels, percent expressed, and distribution across cell types in the transcriptional embedding.

Immune infiltration scores were estimated in bulk samples using ssGSEA (GSVA package, version 1.44.0) with kcdf = “Gaussian”, min.sz = 10, and max.sz = 500, based on cell-type-specific gene signatures constructed from the top 100 marker genes identified by COSG (Section 2.1) (35). Heatmaps and box plots were used to visualize the infiltration scores of different cell types and compare their differences between PAH patients and healthy controls. Correlations among different cell types, as well as between candidate genes and immune infiltration, were assessed using Spearman correlation analysis and visualized with correlation heatmaps.

### 2.9 GSEA-KEGG enrichment analysis for individual candidate genes

For each candidate gene, Pearson correlation coefficients were calculated between its expression and all other genes in the bulk dataset. Genes were ranked in descending order of the correlation coefficient, and GSEA-KEGG enrichment analysis was performed using the gseKEGG function in the clusterProfiler package (version 4.4.0) with the KEGG gene sets (nPerm = 1000, minGSSize = 10, maxGSSize = 1000) (23). Results were visualized using ridge plots.

### 2.10 Gene perturbation and drug prediction

Upstream regulators and downstream targets of the candidate genes were analyzed using the GPSAdb database (version 2.0, https://www.gpsadb.com/), a public resource containing large-scale transcriptomic datasets derived from gene perturbation studies across diverse cell lines (36). For each candidate gene, upstream regulators were defined as genes whose perturbation significantly altered candidate gene expression (|log_2_FC| > 0.5, adjusted *P* < 0.05); downstream targets were defined using the same thresholds. Regulatory networks were constructed using igraph (version 1.4.0) and visualized with ggraph (version 2.1.0).

Drug-gene associations were retrieved from the CTD database (Comparative Toxicogenomics Database, https://ctdbase.org/about/, updated January 29, 2025). A star-shaped network plot was used to visualize the association network between candidate genes and chemical compounds, with red nodes representing candidate genes and blue nodes representing chemical compounds. Drug prediction was performed using the CIGS database (https://cigs.iomicscloud.com/), which contains chemical perturbation data for over 13,000 compounds, including MedChemExpress (MCE) bioactive compounds and traditional Chinese medicines (TCM) (37). For each candidate gene, compounds with adjusted *P* < 0.05 were considered significant; for compounds with multiple concentrations, the concentration with the largest absolute log_2_FC was selected. Drug effects were visualized using bar plots.

### 2.11 Metabolic pathway correlation analysis

Single-cell metabolic pathway scores were calculated using the AddModuleScore function based on KEGG and Reactome gene sets. For each candidate gene, Pearson correlation coefficients were calculated between its expression and the pathway scores. *P*-values were adjusted using the Benjamini-Hochberg (FDR) method. Correlations were visualized in heatmaps.

### 2.12 Transcriptomic clustering analysis

Consensus clustering was performed using ConsensusClusterPlus (version 1.60.0) based on the expression of the 10 candidate genes, using the PAM algorithm with Euclidean distance (reps = 50, pItem = 0.8, pFeature = 1) (38). The optimal number of clusters (k = 2) was determined by the CDF curve. PCA was performed using prcomp with scaling, and the first two principal components were visualized in a scatter plot, with samples colored by cluster. Differences in candidate gene expression between clusters were visualized using box plots, with significance assessed by Student’s t-test. Pathway activity differences were assessed using GSVA (version 1.44.0) with KEGG and Reactome gene sets (min.sz = 10, max.sz = 500). Differential pathway activity between clusters was analyzed using limma (|log_2_FC| > 0.1, adjusted *P* < 0.05), and the top 20 differentially active pathways were visualized in heatmaps. This analysis was exploratory, and the resulting groups should be interpreted as transcriptomic strata rather than clinically validated subtypes. PCA separation based on the same genes used to define the groups does not constitute independent validation.

### 2.13 Animal model and ethical statement

For the animal experiments described below, all procedures were approved by the Medical Ethics Committee of the Second Affiliated Hospital of Fujian Medical University (Approval No. 2024-609, Quanzhou, China) and conducted in accordance with the guidelines outlined in the “Guide for the Care and Use of Laboratory Animals” (8th edition, National Academies Press, 2011). Male Sprague-Dawley rats (200–220 g) were obtained from Sibeifu (Suzhou) Biotechnology Co., Ltd. (certificate of quality SCXK 2022-0006) and maintained under standard conditions. Following our established protocol (39), rats were randomly divided into MCT and control groups (n = 8/group). PAH was induced by a single subcutaneous injection of monocrotaline (MCT, 40 mg/kg; MedChemExpress, Monmouth Junction, NJ, USA); controls received an equal volume of sterile saline. Twenty-eight days post-injection, rats were anesthetized with tribromoethanol (2.5%, 300 mg/kg; Beijing Jitian Biotechnology Co., Ltd., Beijing, China) for hemodynamic examination. Lung tissues were harvested for histological analysis or snap‐frozen at -80 °C for subsequent molecular analyses.

### 2.14 Hemodynamic measurements and histopathological analysis

Hemodynamic measurements were performed following a previously described protocol (39). Briefly, the right jugular vein was isolated through a cervical incision, and a polyethylene catheter filled with heparinized saline was inserted into the right ventricle (RV) via the same vein. The RV systolic pressure (RVSP) was then recorded using a Powerlab-ML221 data acquisition system (AD Instruments Pty Ltd, Bella Vista, New South Wales, Australia). Following hemodynamic assessment, the heart was excised. The RV was dissected and weighed, and the LV along with the IVS was weighed together to calculate the RV hypertrophy index (RVHI) [RV/(LV+IVS)]. Lungs were harvested, fixed in 4% paraformaldehyde, embedded in paraffin, and sectioned at 5 μm for hematoxylin-eosin (H&E) staining. Pulmonary arterioles were photographed, and wall thickness (WT), external diameter (ED), wall area (WA), and total area (TA) were measured using Image-Pro Plus 6.0. Wall thickness (WT%) and wall area (WA%) were then calculated as (2×WT/ED)×100% and (WA/TA)×100%, respectively.

### 2.15 Reverse transcription-quantitative PCR analysis

As described previously (39), total RNA was extracted from whole lung tissues using the FastPure Cell/Tissue Total RNA Isolation Kit V2 (Vazyme, Nanjing, China), cDNA was synthesized using the Hifair^®^ III 1st Strand cDNA Synthesis Kit (Yeasen Biotech, Shanghai, China), and RT-qPCR was performed on the LightCycler^®^ 96 System (Roche Diagnostics, Mannheim, Germany) with the Hieff^®^ qPCR SYBR Green Master Mix (Yeasen Biotech, Shanghai, China). Primers are listed in **Table 1**. Relative gene expression was normalized to *GAPDH* and calculated using the 2^-ΔΔCt^ method.

**Table 1:** Primer sequence for RT-qPCR.

Gene	Forward primer (5′→3′)	Reverse primer (5′→3′)
*RGS1*	ATACTCGGGAATCGACAGCC	AAGGTATTCGCCTGAAGGTCA
*PHLDA1*	ACTGAGGAAGGGCTACTGCT	GCTCCACGCAGTCTACAGTC
*OLR1*	TGAACTTCGTCTTACAAGCAACT	CAGCAAACACAACTCCTCCTT
*PDE4B*	TTCAAGAGGAACATTGCGACAT	GGACATATCAGTTGCTAACACCAT
*MAFB*	CGTCAACGACTTCGACCTTC	AGGTCCTCGAGATGGGTCTT
*PREX1*	GCGTACTCAACGAGATCCTGG	TGACATTCTCTTCCGTGAGGC
*ARRB2*	GTACTTGGGCAAGCGTGACTT	GGTAGGTGGCGATGAACAGAT
*PLEKHO2*	ATCCGTACTGTCCTCAAGCG	GCCTCGGTTGATCCCTTCAT
*GRINA*	CTCTTCATCTTCGCCATACTCTG	GCCGCAAACACATATTCTTCTG
*RNF13*	CAAGGTGACTCGGACTCTGA	CTGCTGTCGGTCTCTTCATTG
*GAPDH*	GACATCAAGAAGGTGGTGAAGC	TGTCATTGAGAGCAATGCCAGC

### 2.16 Statistical analysis

Statistical analyses were performed using R (version 4.2.2). A random seed of 42 was set for reproducibility. For single-cell analyses, donors were treated as biological replicates; cells were not independent observations. Cellular-level comparisons are exploratory. For single-cell differential abundance analyses, including MiloR and cell-count/Fisher/odds-ratio comparisons, the inferential unit was the cell-level neighborhood or cell-type category, not the donor. These analyses are descriptive and exploratory; they do not support donor- level PAH- control inference. Comparisons between two groups were performed using the Wilcoxon rank-sum test or Student’s t-test, as appropriate. Correlations were assessed using Pearson or Spearman correlation, as appropriate. Unpaired Student’s t-test was used for comparisons between MCT and control groups, with normal distribution and homogeneity of variance confirmed prior to analysis. *P* < 0.05 was considered statistically significant, with significance levels indicated as *P < 0.05, **P < 0.01, ***P < 0.001, ****P < 0.0001.

## 3 Results

### 3.1 Single-cell atlas reveals cellular heterogeneity and senescence-associated features in PAH

To dissect the cellular landscape of PAH, scRNA-seq was performed on pulmonary vascular tissues from PAH patients and healthy donors. After quality control and data integration, 14 distinct cell types were identified based on canonical marker genes (**Figures 1A, D**), including B.cells, dendritic cells (DC), endothelial cells (Endo.1, Endo.2), epithelial cells, fibroblasts (Fibro), granulocytes, monocytes/macrophages (Mono.Macs), natural killer cells (NK.cells), smooth muscle cells (SMC.1, SMC.2), T.cells, and T.NK cells (**Figure 1A**). Cells from both PAH patients and healthy donors were distributed across all major cell clusters. Notably, cells from PAH patients showed relatively higher representation in clusters corresponding to DC, Mono.Macs, T.cells, NK cells, and T.NK cells, whereas they were less represented in Fibro clusters (**Figures 1B, C**).

**Figure 1:**
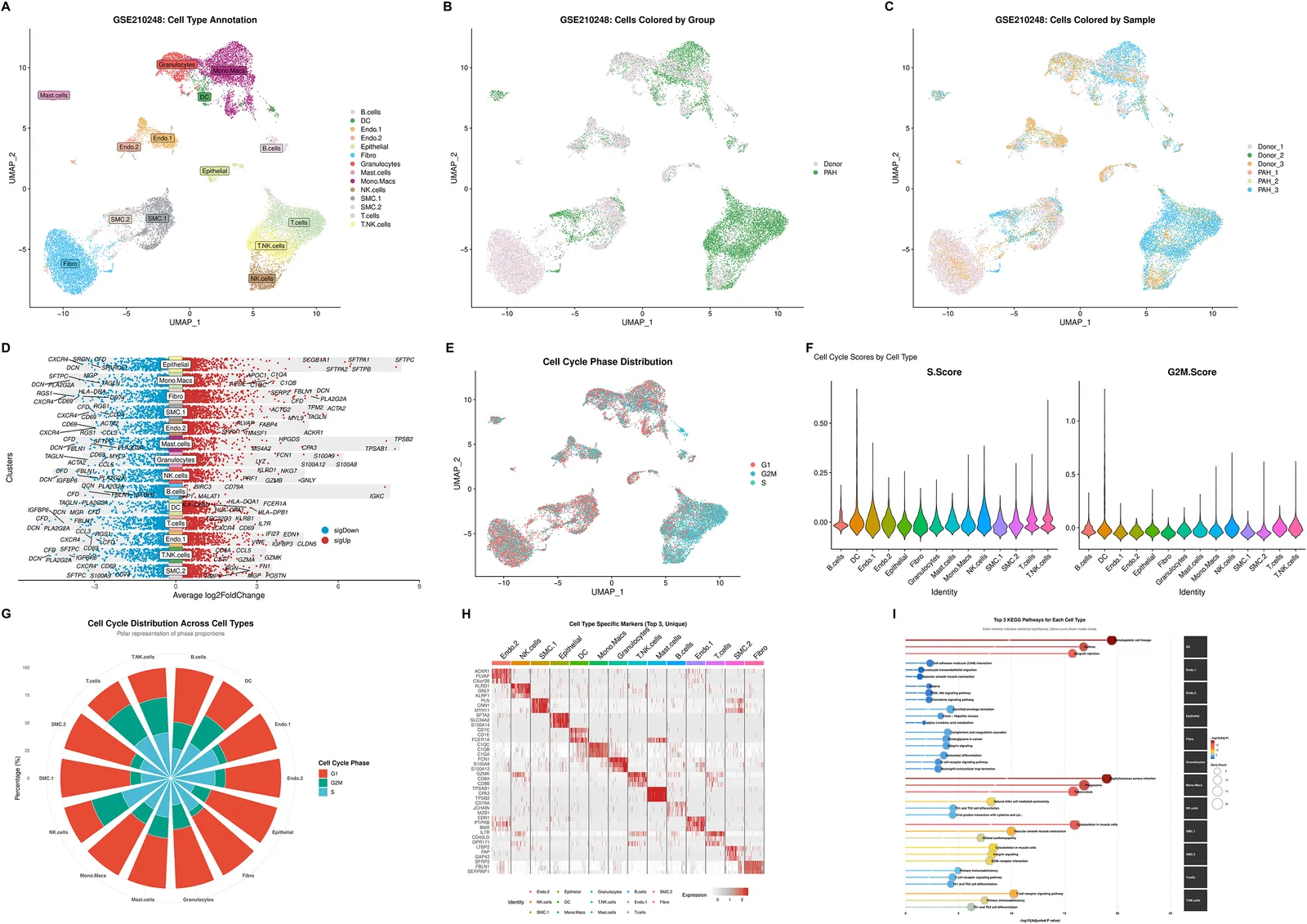
Single-cell transcriptomic landscape and cell annotation. **(A)** UMAP plot showing the distribution of 14 cell types after cell annotation. **(B)** UMAP plot colored by disease group (Donor vs. PAH). **(C)** UMAP plot colored by sample origin, showing the distribution of Donor 1–3 and PAH 1-3. **(D)** Volcano plots displaying differentially expressed marker genes for each cell type. **(E)** UMAP plot colored by cell cycle phase, showing the distribution of cells in G1, S, and G2M phases. **(F, G)** Violin plots and polar plots showing the S.Score and G2M.Score distributions and cell cycle distribution for each cell type. **(H)** Heatmap showing the expression patterns of the top three marker genes for each cell type. **(I)** Lollipop plot showing the top three enriched KEGG pathways for each cell type. Single-cell data were derived from 3 PAH patients and 3 control donors (n = 3 per group).

Cell cycle analysis was subsequently performed across all 14 cell types to assess their proliferative status. The results revealed that while the majority of cells were in the G1 phase, distinct proliferative patterns were observed among specific cell types.

Notably, a subset of immune cells, including T.cells, NK cells, and T.NK cells, exhibited higher S and G2M phase scores, indicating active proliferation. In contrast, structural cells such as epithelial cells, SMC.1, and SMC.2 were predominantly in the G1 phase, suggesting a quiescent state (**Figures 1E–G**). These findings highlight the heterogeneity in proliferative activity across cell types, with specific lymphocyte subsets showing cycling potential while structural cells remain largely non-proliferative.

The robustness of cell annotation was further confirmed by the cluster-specific expression of top marker genes (**Figure 1H**). For example, Mono.Macs highly expressed *C1QC*, *C1QB*, and *C1QA*; T cells were characterized by *IL7R*, *CD40LG*, and *GPR171*; and Fibro showed high expression of *SFRP2*, *FBLN1*, and *SERPINF1*. Additionally, top 3 KEGG pathway enrichment analysis of the top 100 marker genes revealed distinct functional profiles across cell types. Immune cells such as Mono.Macs were enriched in pathways related to staphylococcus aureus infection, phagosome, and tuberculosis, whereas structural cells, notably SMC.1 showed enrichment in pathways associated with cytoskeletal in muscle cells, vascular smooth muscle contraction, and dilated cardiomyopathy (**Figure 1I**). These results support the accuracy of cell annotation and highlight the functional diversity of the identified populations.

To investigate the senescence-associated transcriptional program, a senescence-associated score was calculated for each cell. The highest senescence-associated scores were observed in DC, T cells, and Mono.Macs (**Figure 2A**). To assess the robustness of the senescence score, we compared the original AddModuleScore with an independent UCell rank-based scoring approach using the same gene set. The two scores were highly correlated across Mono.Macs (Spearman rho = 0.804; n = 3,625 cells from six donors) (**Supplementary Figure 1**). Based on the median score, all cells were stratified into senescence-associated score-defined groups (high and low; **Figure 2B**). Comparison of cell abundance between the two groups revealed distinct differences in cell counts and proportions (**Figure 2C**). MiloR differential abundance analysis revealed shifts within transcriptomic neighborhoods, with several cell types exhibiting marked changes in abundance between the two senescence-associated score-defined groups (**Figure 2D**). These results are descriptive and lack donor-level inferential power. Consistently, odds ratio analysis demonstrated that T cells, Mono.Macs, and DC were more frequently represented in the high-score group, whereas epithelial cells, B cells, and SMC.1 were more frequently represented in the low-score group (**Figure 2E**). This analysis is descriptive and should not be interpreted as donor-level PAH-control differential abundance. Together, these results indicate that cell-type composition differs between the two score-defined strata, with immune cells, particularly T cells and Mono.Macs, more frequently represented in the high-score group.

**Figure 2:**
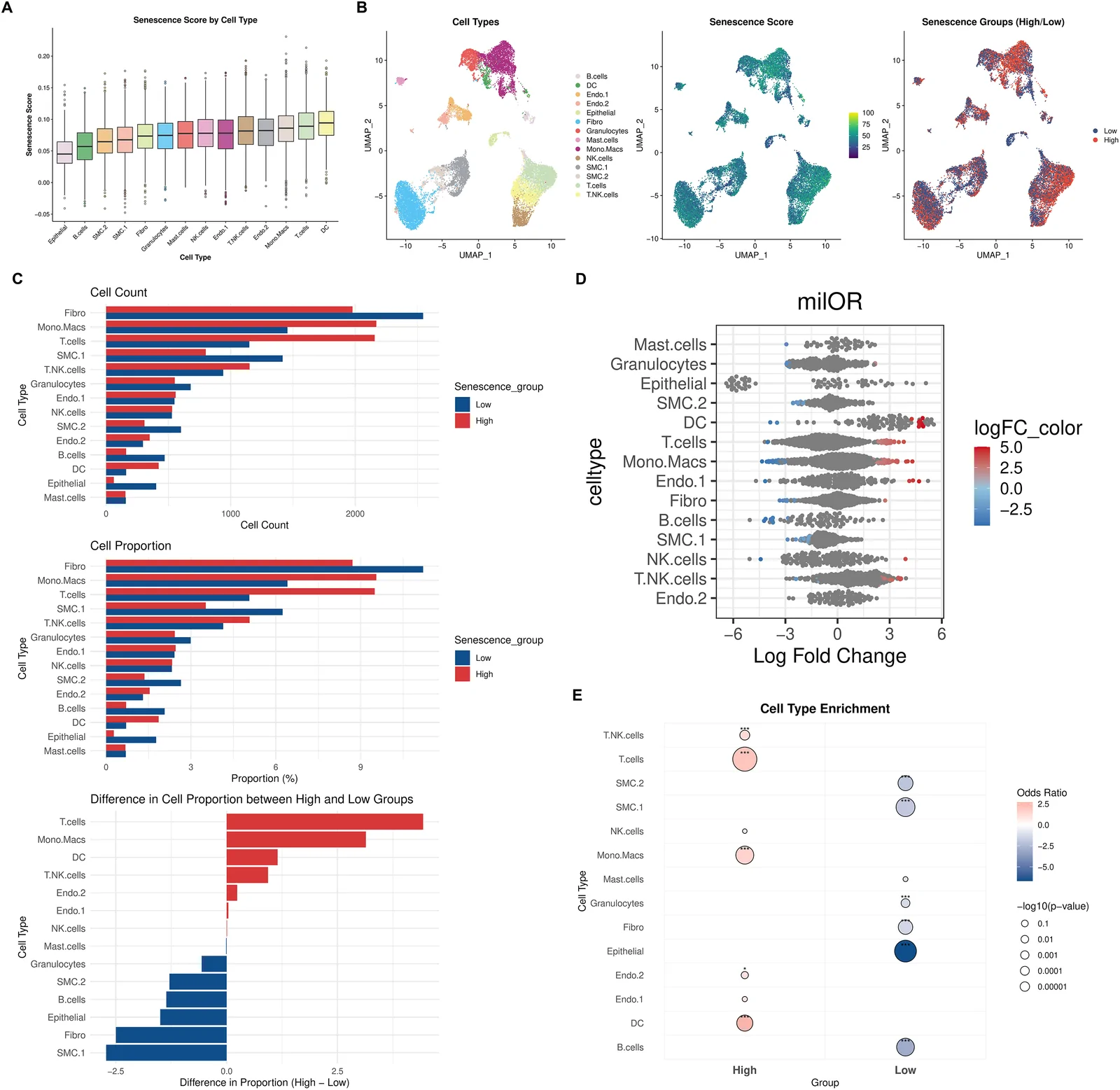
Senescence score and differential cell abundance analysis. **(A)** Box plots showing the senescence scores of each cell type. **(B)** Triple UMAP plots showing cell type distribution, distribution of senescence-associated scores in the transcriptional embedding, and the high-/low- senescence-associated score groups. **(C)** Bar plots comparing the count, proportion, and difference of each cell type between the high- and low- senescence-associated groups. **(D)** MiloR beeswarm plot showing differential abundance results at the neighborhood level (logFC) for each cell type, with colors indicating the direction and magnitude of significant changes. **(E)** Odds ratio bubble plot showing the enrichment of each cell type in the high- and- low senescence-associated groups. These analyses are descriptive and lack donor-level inferential power. MiloR differential abundance was tested at the neighborhood level; odds ratios were calculated using Fisher’s exact test; donors are the biological replicates (n = 3 per group).

### 3.2 Senescence-associated transcriptional signature is linked to stemness, pathway activity, and intercellular communication

To further characterize the functional consequences of senescence, cellular stemness was assessed using CytoTRACE analysis. The distribution in the transcriptional embedding of stemness scores across all cells was visualized by UMAP (**Figure 3A**). Among all cell types, epithelial cells, SMC.1, and SMC.2 exhibited the highest CytoTRACE scores, whereas T cells, T.NK cells, and NK cells displayed the lowest (**Figure 3B**). Notably, cells in the high senescence-associated score group exhibited lower stemness scores compared to those in the low-score group (**Figure 3C**). These results are descriptive and do not support donor-level inference. Analysis of mean CytoTRACE scores further revealed that epithelial cells, SMC.2, and Fibro maintained consistently high scores in both score groups, while NK cells, T.NK cells, and T cells remained low across both groups (**Figure 3D**). Pathway activity was further analyzed to assess the functional impact of these senescence-associated changes. Waterfall plot analysis of Hallmark pathways showed that Peroxisome, KRAS signaling up, and Hypoxia exhibited higher scores in the high senescence-associated score group, whereas Mitotic spindle, Apoptosis, and Estrogen response late exhibited lower scores (**Figure 3E**). Similarly, marked differences in metabolic pathway scores were observed between the two groups based on KEGG and Reactome databases (**Figure 3F**). Together, these results align with the reduced stemness in cells with high senescence-associated scores, suggesting that senescence-associated transcriptional signature is linked to altered differentiation potential and metabolic reprogramming.

**Figure 3:**
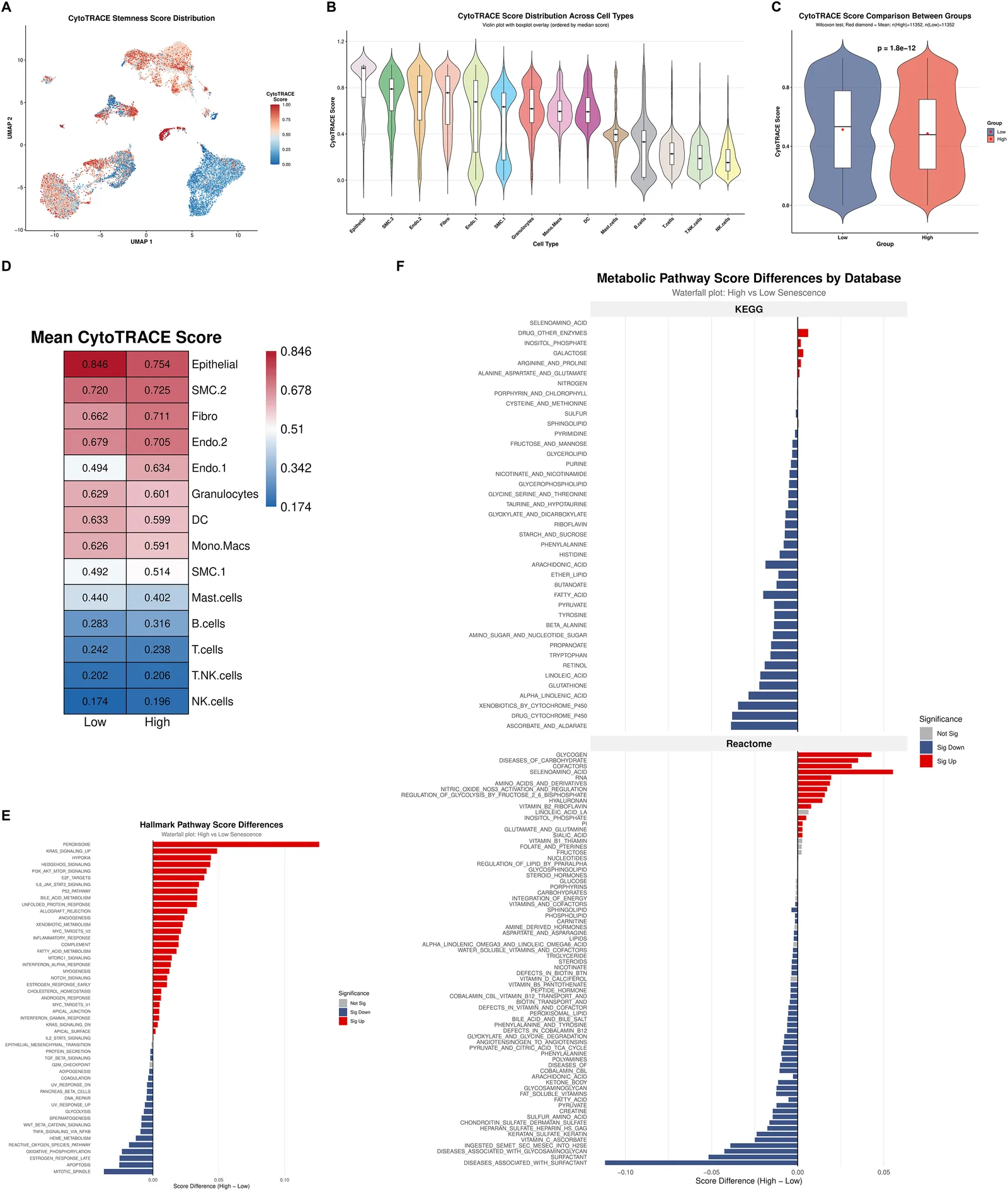
Stemness and pathway activity analysis. **(A)** UMAP plot showing the distribution of CytoTRACE stemness scores in the transcriptional embedding. **(B)** Violin plots showing the distribution of CytoTRACE scores across cell types. **(C)** Violin plot comparing CytoTRACE scores between the high- and low-senescence-associated score groups; the *P* value was calculated at the cell level and does not constitute donor-level statistical inference. **(D)** Heatmap showing the mean CytoTRACE score of each cell type in the high- and low-senescence-associated groups. **(E)** Waterfall plot displaying the differences in Hallmark pathway scores between the high- and low- senescence-associated groups. **(F)** Waterfall plots showing the differences in metabolic pathway score between groups based on KEGG and Reactome databases, respectively. CytoTRACE and pathway score comparisons are descriptive; no donor-level statistical inference was performed. n = 3 per group.

Given these functional changes, intercellular communication was next examined. CellChat analysis inferred communication patterns associated with the high senescence-associated score group, with a higher total number of interactions and greater interaction strength compared to the low-score group (**Figures 4A, B**).

**Figure 4:**
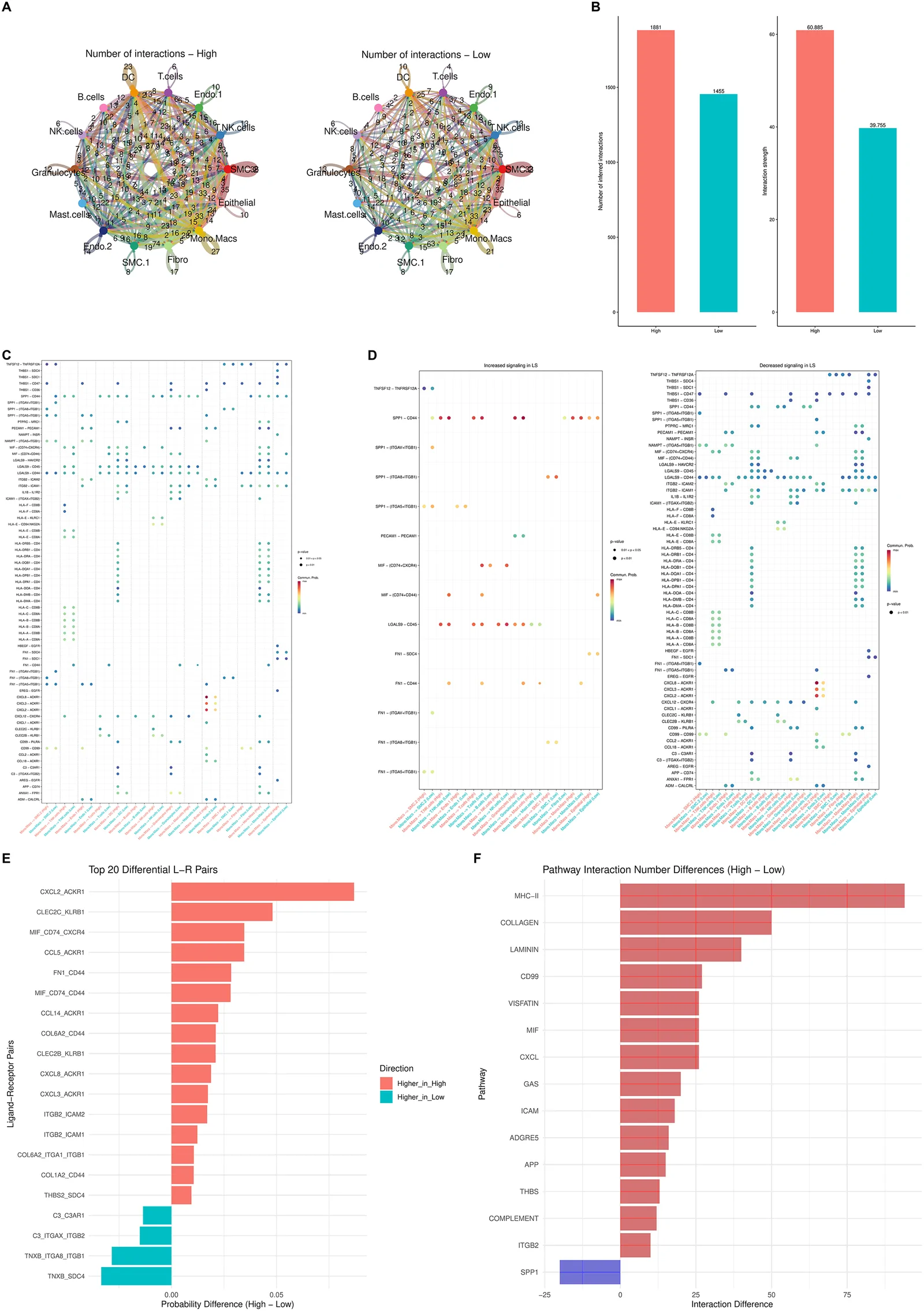
Cell communication analysis. **(A)** Network plots showing the number of interactions among cell types in the high- and low- senescence-associated groups. **(B)** Bar plots showing the total number of inferred interactions (left) and the interaction strength (right) in the two groups. **(C)** Dot plot illustrating ligand-receptor pairs with altered signaling between the two groups. **(D)** Bubble plots showing ligand-receptor pairs that were enhanced or weakened in the low senescence-associated score group. **(E)** Bar plot displaying the top 20 ligand-receptor pairs with the greatest differences. **(F)** Bar plot showing differences in the number of interactions for each signaling pathway. CellChat results represent inferred communication patterns associated with the senescence-associated score. Composition and cell-number sensitivity analyses were not performed; these results are exploratory. n = 3 per group.

Several signaling pathways showed altered inferred activity between the two groups (**Figure 4C**), and specific ligand-receptor pairs differentially inferred in the low-score group were identified via bubble plots (**Figure 4D**). The top 20 differentially inferred ligand-receptor pairs were displayed in a bar plot (**Figure 4E**). Notably, pairs including CXCL2-ACKR1, CLEC2C-KLRB1, and MIF-CD74-CXCR4 were more prominent in the inferred communication of the high-score group, whereas TNXB-SDC4, TNXB-ITGA8-ITGB1, and C3-ITGAX-ITGB2 were more prominent in the low-score group. Further quantification of inferred signaling pathway interaction counts showed that MHC-II, COLLAGEN, and LAMININ pathways exhibited the most pronounced differences in the high-score group (**Figure 4F**). Collectively, these findings suggest that the high senescence-associated score group is characterized by globally altered inferred intercellular communication, predominantly involving immune-related and extracellular matrix signaling pathways. Composition and cell-number sensitivity analyses were not performed for CellChat inference; therefore, these results should be interpreted as descriptive and exploratory.

### 3.3 Integrative analysis identifies Mono.Macs-associated senescence-related candidate genes

To identify senescence-associated genes enriched in Mono.Macs, a two-step screening strategy was implemented. The intersection of the 1,853 genes identified by correlation filtering and the 1,132 genes identified by differential expression filtering yielded 405 candidate genes (**Figure 5A**). These genes were highlighted as the “final candidate gene set” in the volcano-style scatter plot (**Figure 5B**). Bubble plot analysis revealed that the top 20 candidate genes were predominantly expressed in Mono.Macs, granulocytes, and DC (**Figure 5C**). Moreover, the top 20 candidate genes were significantly enriched in Mono.Macs (**Figure 5D**). Notably, chemokines such as *CXCL8*, *CXCL2*, and *CCL3* were particularly highly expressed in Mono.Macs compared with other cell types.

**Figure 5:**
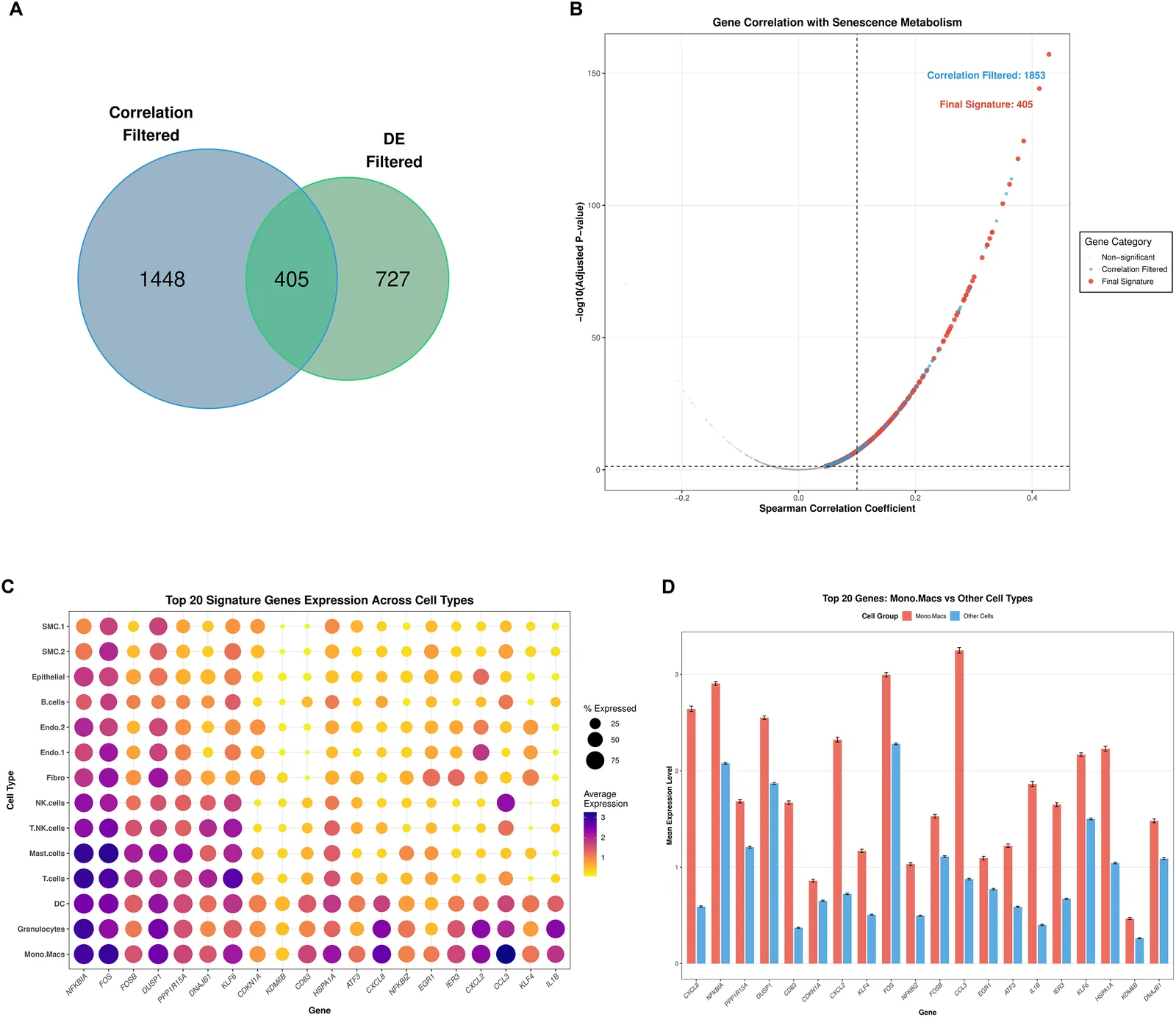
Identification of Mono.Macs senescence-associated genes. **(A)** Venn diagram showing the intersection of genes identified by correlation and DE filters. **(B)** Volcano-style scatter plot displaying the Spearman correlation coefficients and adjusted P-values for all genes. **(C)** Bubble plot showing the expression patterns of the top 20 candidate genes across different cell types. **(D)** Bar plot comparing the mean expression levels of the top 20 genes in Mono.Macs versus other cells. Single-cell data were derived from 3 PAH patients and 3 control donors (n = 3 per group). *P* values in panel B were adjusted using the Benjamini-Hochberg method.

To examine these single-cell findings at the tissue level, three bulk transcriptomic datasets were analyzed. Distinct batch effects were observed across the datasets before correction; after batch effect correction, the samples were well integrated (**Figures 6A, B**). A total of 1,469 DEGs were identified between PAH patients and healthy controls, with 34 genes overlapping the Mono.Macs-associated candidate genes highlighted in the volcano plot (**Figure 6C**). The expression patterns of top DEGs were visualized in a heatmap (**Figure 6D**). The intersection between the 790 up-regulated and 679 down-regulated DEGs with the 405 Mono.Macs-associated senescence-related candidate genes was illustrated by a Venn diagram, revealing 11 and 23 overlapping genes, respectively (**Figure 6E**). Enrichment results for all DEGs and the intersecting DEGs in GO (BP, CC, MF) and KEGG pathways are shown in **Figures 6F, G**, respectively. GO analysis revealed significant enrichment in immune-related processes, including cell chemotaxis (BP), tertiary granule (CC), and cytokine receptor binding (MF), while KEGG analysis further identified rheumatoid arthritis as a significantly enriched pathway (**Figure 6G**).

**Figure 6:**
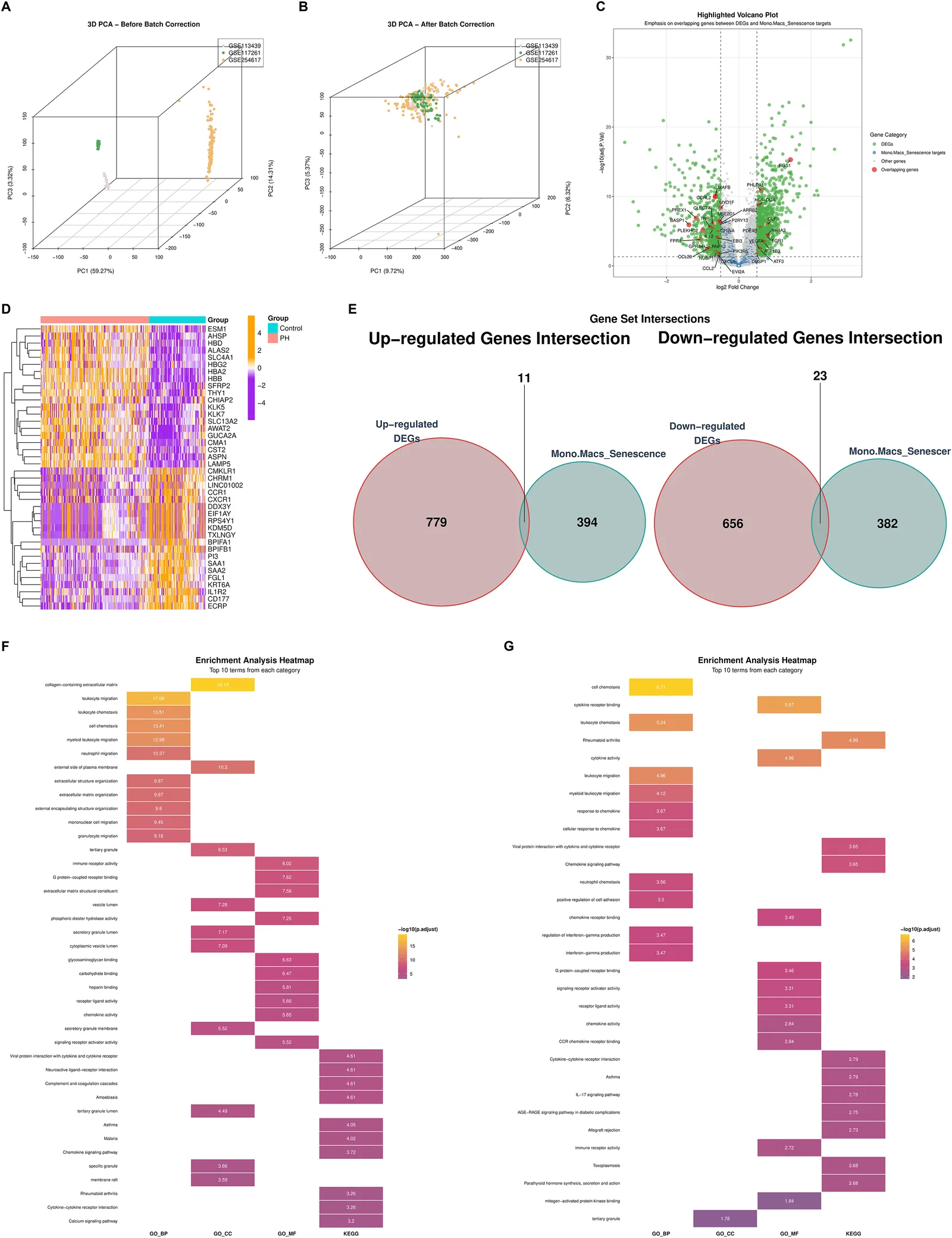
Bulk transcriptomic profiling identifies Mono.Macs senescence-related candidate gene set in PAH. **(A, B)** 3D PCA plots showing the distribution of samples from three bulk transcriptomic datasets (n = 257 expression profiles from 189 unique subjects) before **(A)** and after **(B)** batch effect correction. Each color represents a different dataset. **(C)** Volcano plot of DEGs between PAH and Controls (|log_2_FC| > 0.5, adjusted *P* < 0.05). Red dots indicate genes overlapping with the Mono.Macs-associated senescence-related candidate genes identified by our screening strategy. **(D)** Heatmap showing the expression patterns of top DEGs between PAH and controls. Orange indicates high expression; purple indicates low expression. **(E)** Venn diagram illustrating the intersections of up-regulated and down-regulated DEGs with the Mono.Macs senescence-related gene set. **(F, G)** Heatmaps showing the enrichment results of all DEGs **(F)** and the intersecting DEGs **(G)** in GO (BP, CC, MF) and KEGG pathways, respectively. The color scale represents the normalized enrichment score.

To refine the candidate list to the most robust candidate, three machine learning algorithms—LASSO regression, Random Forest, and XGBoost—were applied. The expression differences of the 34 candidate genes between PAH and control groups were visualized by violin plots (**Figure 7A**). The cross-validation curve of LASSO regression was used to illustrate the selection of the optimal lambda value (**Figure 7B**). The Random Forest model ranked *RGS1*, *HLA-DOA*, and *UBE2D1* as the top three genes (**Figure 7C**), while the XGBoost model identified *RGS1*, *MAFB*, and *PLEKHO2* as the top three features (**Figure 7D**). The intersection of the three algorithms yielded 10 prioritized candidate genes: *ARRB2*, *GRINA*, *MAFB*, *OLR1*, *PDE4B*, *PHLDA1*, *PLEKHO2*, *PREX1*, *RGS1*, and *RNF13* (**Figure 7E**). The chord diagram revealed predominantly positive correlations among the 10 candidate genes, with only a few negative associations (**Figure 7F**). ROC curves were generated to evaluate the discovery-stage discriminatory capacity of the prioritized candidate genes. Among the 10 candidate genes, *RGS1* exhibited the highest AUC (AUC = 0.818, 95% CI: 0.764-0.873), followed by *PLEKHO2* (AUC = 0.773, 95% CI: 0.712-0.834) and *PHLDA1* (AUC = 0.765, 95% CI: 0.706-0.824) (**Figure 7G**). The three-algorithm intersection was used to prioritize candidate genes rather than to develop a clinical prediction model.

**Figure 7:**
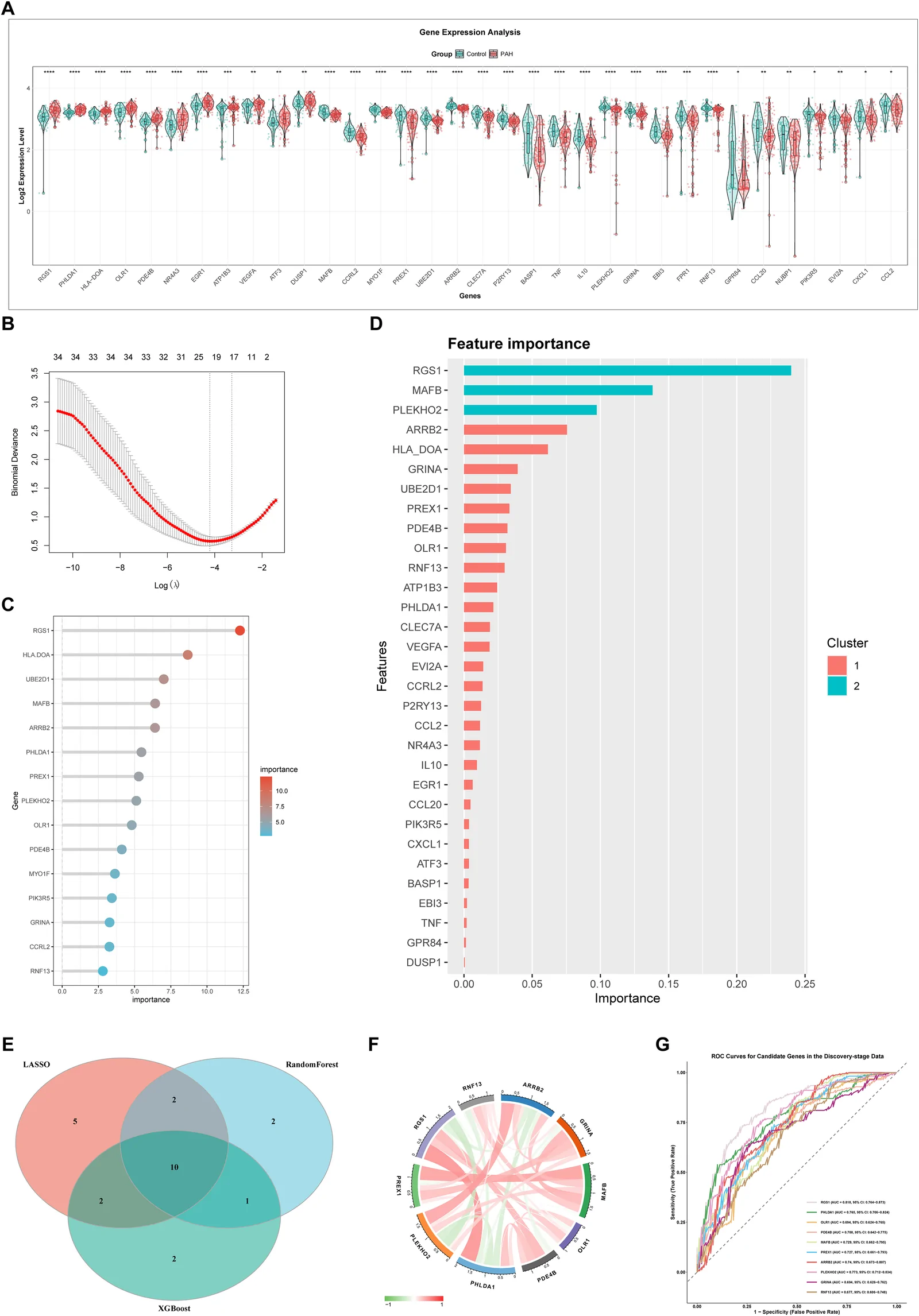
Prioritization of candidate genes via machine learning. **(A)** Violin plot showing the expression differences of the 34 candidate genes between the PAH and Control groups. **(B)** Cross-validation curve of LASSO regression illustrating the selection process of the optimal lambda value. **(C)** Gene importance ranking based on the Random Forest model. **(D)** Feature importance ranking from the XGBoost model. **(E)** Venn diagram showing the intersection of genes identified by LASSO, Random Forest, and XGBoost, yielding 10 candidate genes. **(F)** Chord diagram illustrating the correlations among the 10 candidate genes. **(G)** ROC curves assessing the discovery-stage discriminatory capacity of each candidate gene. Notably, this figure shows candidate prioritization results from the discovery-stage data and does not represent performance of a clinical prediction model. For panel A, limma moderated t-test with Benjamini-Hochberg adjustment was used. n = 257 expression profiles from 189 unique subjects. *P < 0.05, **P < 0.01, ***P < 0.001, ****P < 0.0001.

To further assess the robustness of the prioritized candidates, we examined whether the association between each candidate gene and the senescence score was consistent across donors. Within Mono.Macs from each of the six donors, all 10 candidate genes showed positive correlations with the UCell score (**Supplementary Figure 2**), supporting the robustness of the gene-score associations at the individual level. At the bulk transcriptomic level, to address the non-independence of repeated PHBI subjects between GSE117261 and GSE254617, we performed a sensitivity analysis restricted to 189 unique subjects (120 PAH, 69 control). All 10 prioritized candidate genes retained their discovery-stage direction of change, and all remained significant after within-gene-set BH correction (q < 0.05) (**Supplementary Figure 3**). In the main sensitivity branch with TMM normalization, the same direction was retained, with q values ranging from 1.97×10–^12^ to 0.044. In the alternative prespecified retention rule favoring GSE117261 profiles, all 10 genes retained their direction, with 8 remaining significant; PLEKHO2 and RNF13 showed attenuated effects (q = 0.637 and q = 0.677, respectively). Complete results for both retention rules are provided in **Supplementary Table 1**. This analysis represents a sensitivity test of a fixed candidate set rather than an independent rediscovery. Sensitivity analyses restricted to 189 unique subjects yielded qualitatively consistent results for GSEA, ssGSEA, and consensus clustering. For consensus clustering, k = 2 still yielded two groups, although membership was not entirely identical.

To evaluate the reproducibility of the prioritized candidates, we tested their expected expression directions in independent PAH cohorts that were not used in the discovery phase (**Supplementary Figures 4A–G**). With nominal *P* values, *RGS1* was significantly upregulated in GSE48149 and GSE53408 (both lung), and *PHLDA1* was upregulated in GSE48149 (lung). *MAFB* was significantly downregulated in GSE33463 (PBMC) and GSE48149 (lung), whereas *RNF13* was downregulated in GSE15197 and GSE48149 (both lung). *RGS1* was significantly upregulated in GSE48149 (*P* = 0.0091) and GSE53408 (*P* = 0.0223). *PHLDA1* was significantly upregulated in GSE48149 (*P* = 0.0015). *MAFB* was significantly downregulated in GSE33463 (*P* = 0.00066) and GSE48149 (*P* = 0.0169). *RNF13* was significantly downregulated in GSE15197 (*P* = 5.00×10^-5^) and GSE48149 (*P* = 0.0145). These results reproduced the discovery-stage expression directions for *RGS1*, *PHLDA1*, *MAFB*, and *RNF13* in one or more independent PAH datasets, supporting their prioritization for further biological investigation. After within-cohort multiple-testing correction (BH-adjusted q < 0.05), seven gene-cohort combinations retained directional consistency with the discovery-stage findings, including *RGS1*, *PHLDA1*, *MAFB*, and *RNF13* in lung tissue cohorts, and *MAFB* in a *PBMC* cohort (**Supplementary Figure 5**). The complete 70 gene-cohort results, including nonsignificant and discordant findings, are provided in **Supplementary Table 2**. **Supplementary Figure 4** shows the independent cohort results with nominal *P* values, and **Supplementary Figure 5** shows the subset that remained significant after within-cohort multiple-testing correction.

### 3.4 Candidate genes link to inflammation, immune infiltration, and metabolism

To examine the expression patterns of the 10 candidate genes at the single-cell level, bubble plots were used to visualize their average expression levels and percent expressed across different cell types. These genes exhibited relatively high expression in Mono.Macs, granulocytes, and DC (**Figure 8A**). Among them, *MAFB, ARRB2*, and *RNF13* showed particularly high average expression and percent expressed in Mono.Macs. The distribution of each candidate gene in the transcriptional embedding was further visualized by UMAP plots, confirming their predominant enrichment in Mono.Macs (**Figure 8B**). The expression patterns of genes positively correlated with each candidate gene across all samples are shown in **Supplementary Figures 6A–J**, revealing distinct expression profiles between PAH and control groups.

**Figure 8:**
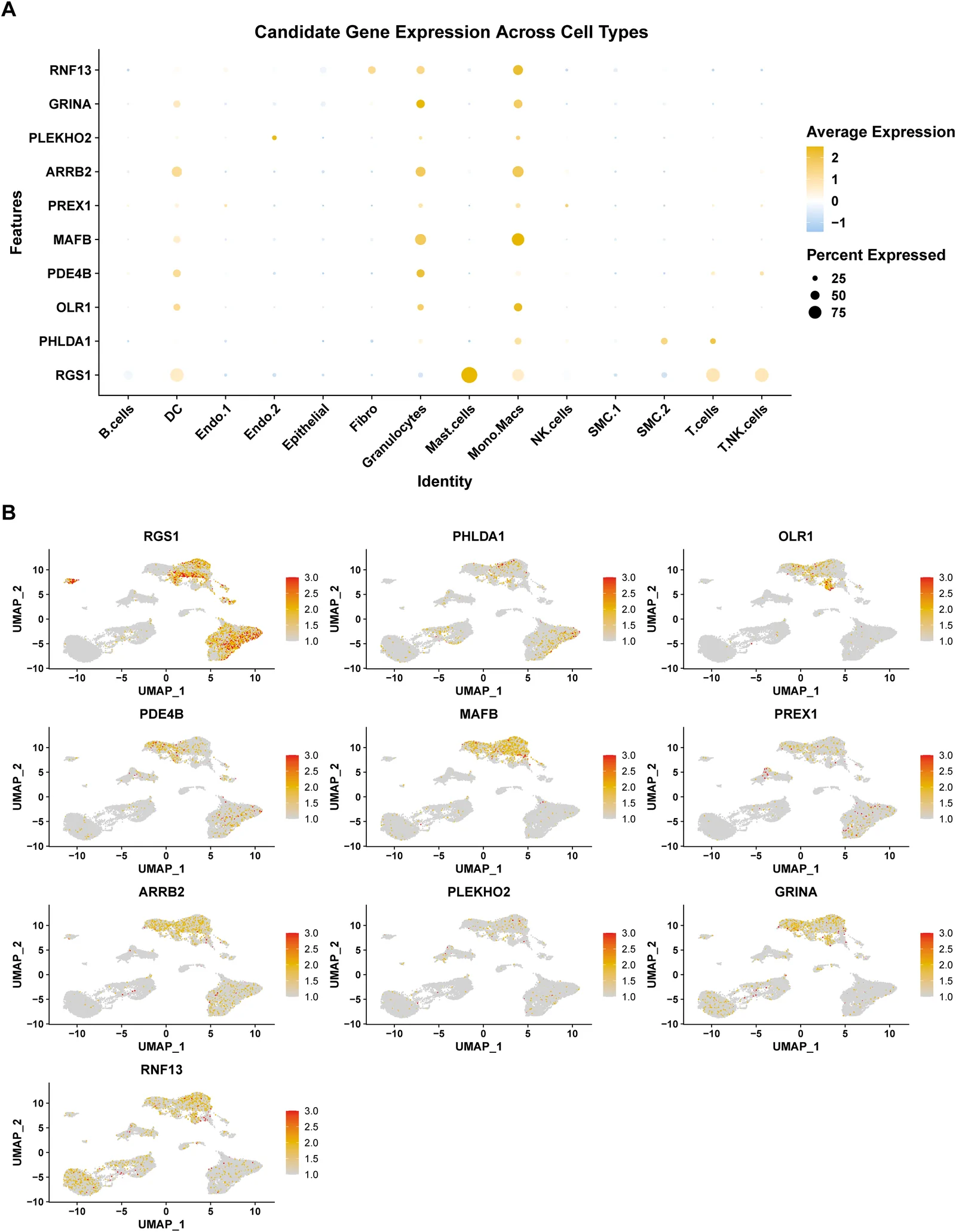
Expression patterns of candidate gene expression in single-cell transcriptomic data. **(A)** Bubble plot showing the expression levels and percentages of the 10 candidate genes across different cell types. Dot color represents the average expression level; dot size represents the percent expressed. **(B)** UMAP plots showing the projection and expression of each candidate gene across different cell clusters in PAH. Color intensity indicates expression level. Data are from 3 PAH patients and 3 control donors (n = 3 per group).

To characterize the cellular landscape of PAH, ssGSEA was performed to evaluate the infiltration scores of various cell types. Distinct infiltration patterns across samples were observed, as shown in the heatmap (**Figure 9A**). Descriptive differences in infiltration scores of various cell types were observed between the PAH and control group. Specifically, the ssGSEA enrichment score for Mono.Macs was lower in PAH patients (**Figure 9B**). Pairwise correlations among cell types were illustrated by a correlation heatmap. Notably, Mono.Macs showed positive correlations with DC, granulocytes, and B.cells, while negative correlations were observed with Endo.1, Endo.2, and SMC.1 (**Figure 9C**). Moreover, Spearman correlation analysis revealed that most candidate genes, including *RNF13*, *GRINA*, *PLEKHO2*, *ARRB2*, *MAFB*, and *OLR1*, were significantly positively correlated with Mono.Macs infiltration, whereas *PHLDA1* showed a significant negative correlation (**Figure 9D**).

**Figure 9:**
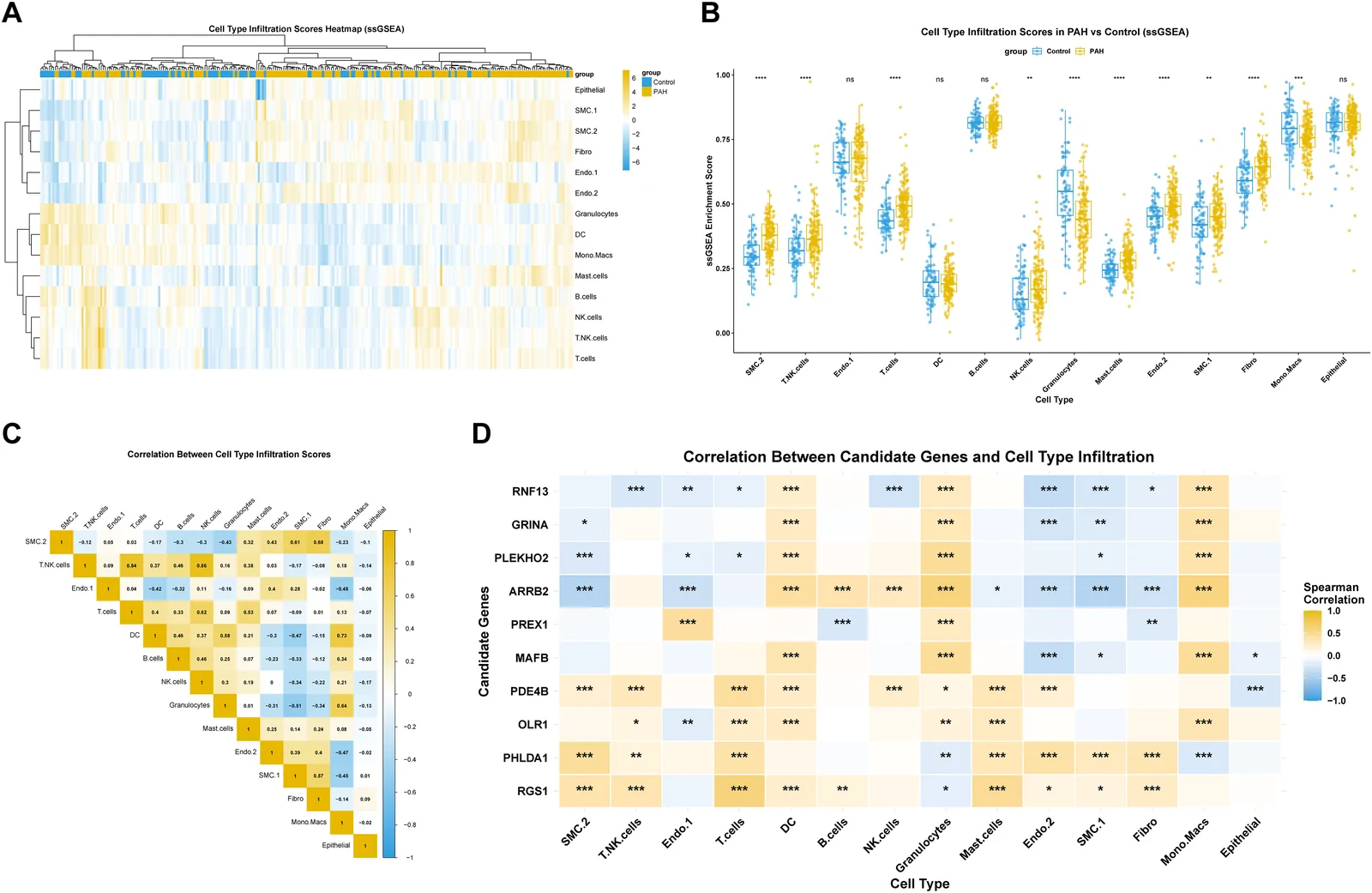
Immune infiltration landscape in PAH and its association with candidate genes. **(A)** Heatmap showing the ssGSEA infiltration scores of different cell types across all samples. **(B)** Box plots comparing the infiltration scores of various cell types between PAH and Control groups. **(C)** Correlation heatmap illustrating the pairwise correlations among the infiltration scores of different cell types. **(D)** Correlation heatmap showing the Spearman correlation coefficients between the 10 candidate genes and the infiltration scores of each cell type. Spearman correlation was used for pairwise correlation analyses. n = 257 expression profiles from 189 unique subjects. *P < 0.05, **P < 0.01, ***P < 0.001.

To explore the biological functions of the 10 candidate genes, GSEA-KEGG enrichment analysis was performed for each candidate gene based on genes ranked by correlation with its expression. Distinct pathway enrichment patterns were observed among the candidate genes (**Figures 10A–J**). Multiple candidate genes, including *ARRB2* and *MAFB*, were significantly enriched in pathways related to macrophage function, such as phagosome, Fc gamma R-mediated phagocytosis, and NF-κB signaling (**Figures 10A, C**). In contrast, *RNF13* showed significant enrichment in pathways associated with cell proliferation and genomic stability, including cell cycle, homologous recombination, and cellular senescence (**Figure 10J**).

**Figure 10:**
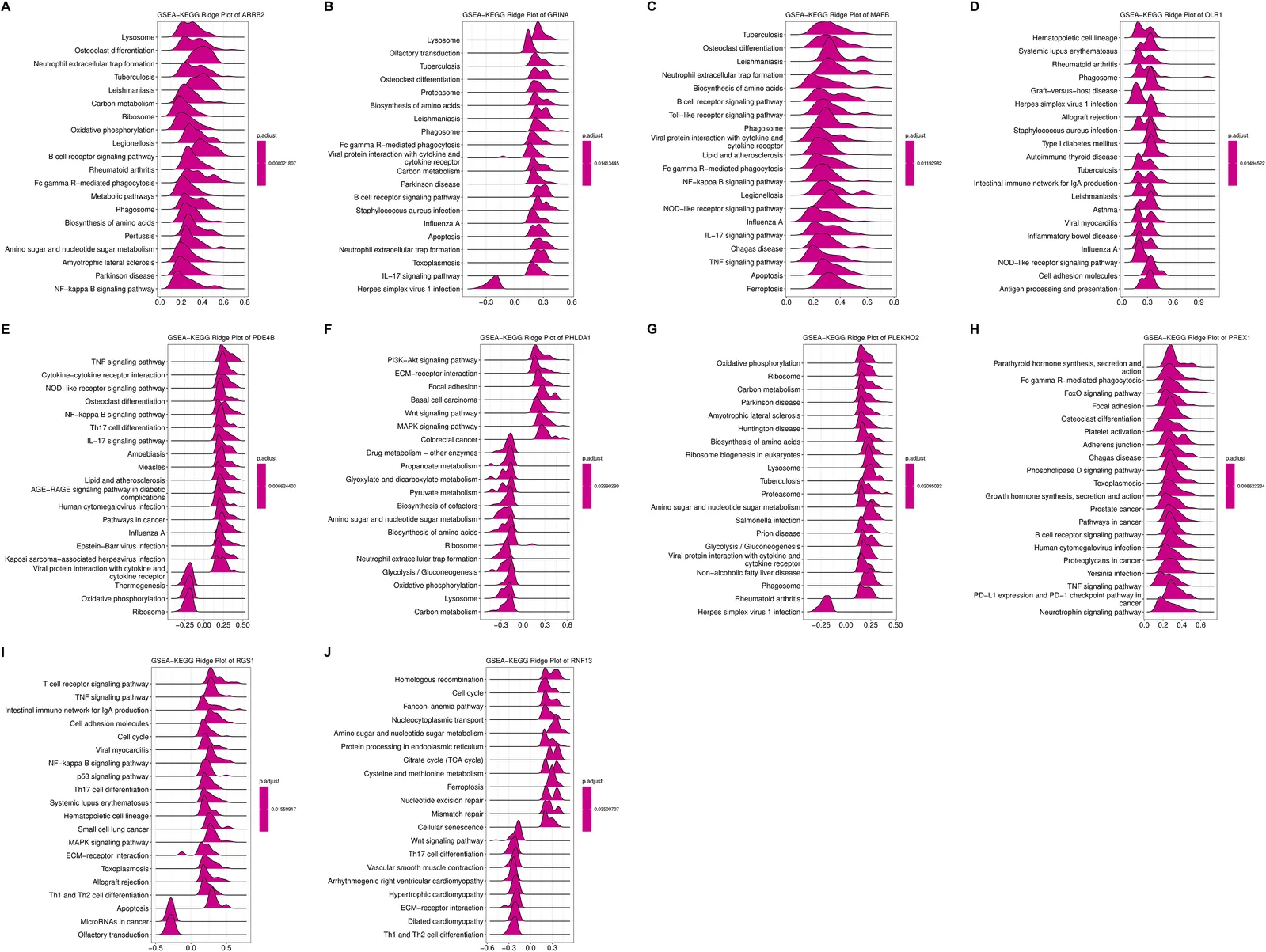
GSEA‐KEGG enrichment analysis for individual candidate genes. **(A–J)** GSEA‐KEGG enrichment plots showing the enriched pathways for each of the 10 candidate genes. GSEA-KEGG enrichment was performed on the discovery-stage bulk transcriptomic data with Benjamini-Hochberg adjustment. n = 257 expression profiles from 189 unique subjects.

The regulatory landscape and potential therapeutic targets of the candidate genes were investigated by analyzing their upstream regulators and downstream target genes. The upstream regulatory network of the candidate genes was illustrated by circular network plot, with red and blue edges indicating activating and inhibitory relationships, respectively (**Figure 11A**). The top upstream regulators for each candidate gene and their effect directions were displayed in a bar plot (**Figure 11B**).

**Figure 11:**
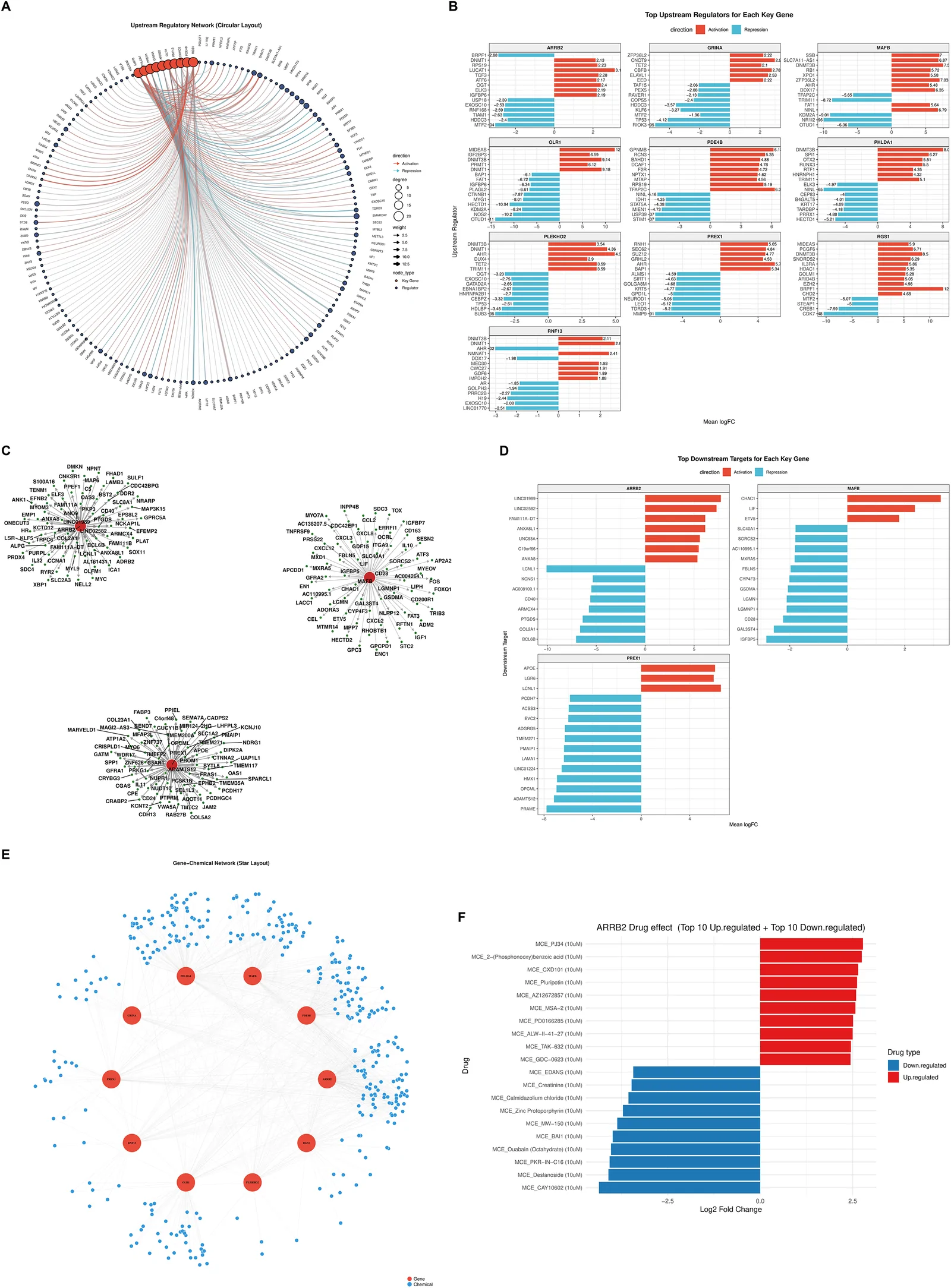
Exploratory gene perturbation and drug prediction analysis. **(A)** Circular network plot illustrating the upstream regulatory network of the candidate genes. Red edges indicate activating relationships; blue edges indicate inhibitory relationships. **(B)** Bar plots displaying the top upstream regulators for each candidate gene and their effect directions. **(C)** Network plot showing the distribution of downstream target genes of the candidate genes. **(D)** Bar plots presenting the top downstream target genes for selected candidate genes. **(E)** Star-shaped network plot showing the associations between candidate genes and chemical compounds based on the CTD database. Red nodes represent candidate genes; blue nodes represent chemical compounds. **(F)** Bar plots depicting drug effects on *ARRB2* expression, including the top 10 compounds predicted to upregulate or downregulate its expression. These analyses are exploratory predictions based on public databases; no experimental validation was performed.

The downstream target genes of selected candidate genes, such as *ARRB2*, *MAFB*, and *PREX1*, were visualized in a network plot, and their top downstream targets were presented in a bar plot (**Figures 11C, D**). For *ARRB2*, *LINC01989* was identified as an activated target, whereas *LCNL1* was repressed. Similarly, for *MAFB*, *CHAC1* was identified as an activated target, whereas *IGFBP5* was repressed (**Figure 11D**). Based on the CTD database, potential drugs targeting the candidate genes were predicted, with detailed results provided in **Supplementary Table 3**. The associations between candidate genes and chemical compounds were visualized as a star-shaped network plot (**Figure 11E**). Among the candidate genes, *ARRB2* was the only one with available perturbation data (PMID: 40826206). Bar plots showed PJ34 and CAY10602 as the top compounds predicted to upregulate and downregulate *ARRB2* expression, respectively (**Figure 11F**).

Single-cell metabolic pathway scores were calculated based on KEGG and Reactome gene sets. The correlations between the 10 candidate genes and KEGG metabolic pathway scores are shown in a heatmap, with significant correlations indicated by asterisks (**Figure 12A**). Positive correlations with porphyrin and chlorophyll metabolism, fructose and mannose metabolism, arginine and proline metabolism, sphingolipid metabolism, and alanine, aspartate and glutamate metabolism were observed for *GRINA*, *RNF13*, *ARRB2*, *PLEKHO2*, *OLR1*, and *MAFB*. In contrast, negative correlations with taurine and hypotaurine metabolism, propanoate metabolism, tyrosine metabolism, retinol metabolism, ether lipid metabolism, linoleic and alpha-linolenic acid metabolism were observed for *RGS1*, *ARRB2*, *PDE4B*, *PHLDA1*, *PREX1*, *OLR1*, and *MAFB* (**Figure 12A**). Similarly, the correlations between the 10 candidate genes and Reactome metabolic pathway scores are shown in a heatmap (**Figure 12B**).

**Figure 12:**
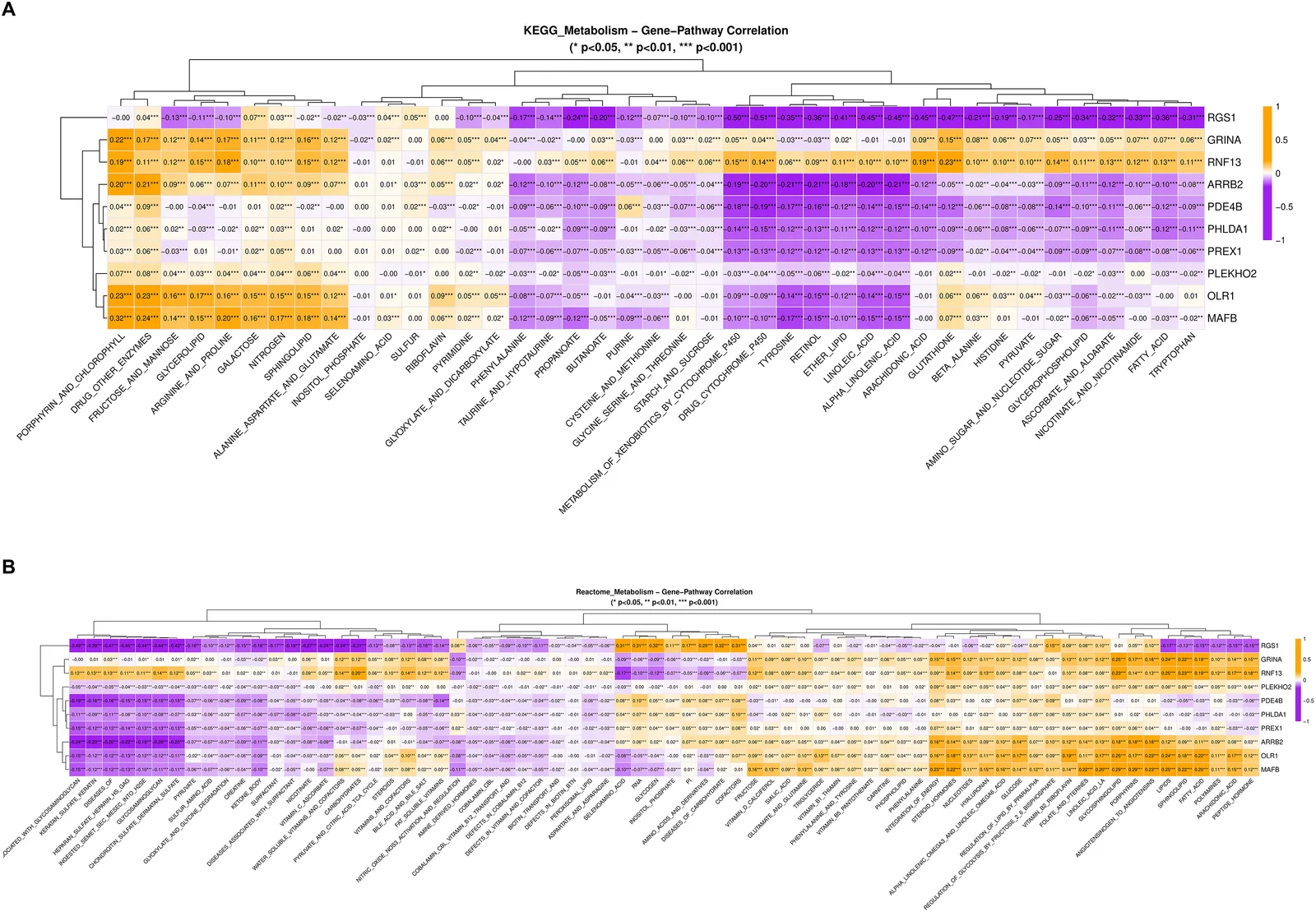
Association between candidate genes and metabolic pathways. **(A)** Heatmap showing the correlations between the 10 candidate genes and KEGG metabolic pathway scores. **(B)** Heatmap showing the correlations between the 10 candidate genes and Reactome metabolic pathway scores. Pearson correlation was used to assess gene-pathway associations, with P-values adjusted using the Benjamini-Hochberg (FDR) method. n = 3 per group.

### 3.5 Candidate genes stratify PAH into distinct exploratory transcriptomic groups

To investigate whether the 10 candidate genes could stratify PAH patients into exploratory transcriptomic subgroups, consensus clustering was performed on the bulk transcriptomic samples using these genes. The consensus matrix heatmap for k = 2 showed clear separation between two distinct clusters, designated as Group A and Group B (**Figure 13A**). PCA displayed the distribution of the two groups in the principal component space (**Figure 13B**). Significant differences in the expression levels of the candidate genes were observed between the two groups, as illustrated by box plots (**Figure 13C**). Notably, compared with Group A, the expression levels of *RGS1*, *PHLDA1*, *OLR1*, *MAFB*, *PREX1*, and *GRINA* were significantly higher in Group B. Moreover, pathway activity analysis revealed distinct functional profiles between the two groups, as shown in the heatmaps of KEGG and Reactome pathway activities (**Figures 13D, E**). In the KEGG analysis, Group B showed significant enrichment of immune-related pathways, including Fc gamma R-mediated phagocytosis, Toll-like receptor signaling pathway, and JAK-STAT signaling pathway (**Figure 13D**). Similarly, in the Reactome analysis, Group B showed significant enrichment of immune-related pathways, including Fcgamma receptor-dependent phagocytosis, interferon gamma signaling, and the role of phospholipids in phagocytosis (**Figure 13E**).

**Figure 13:**
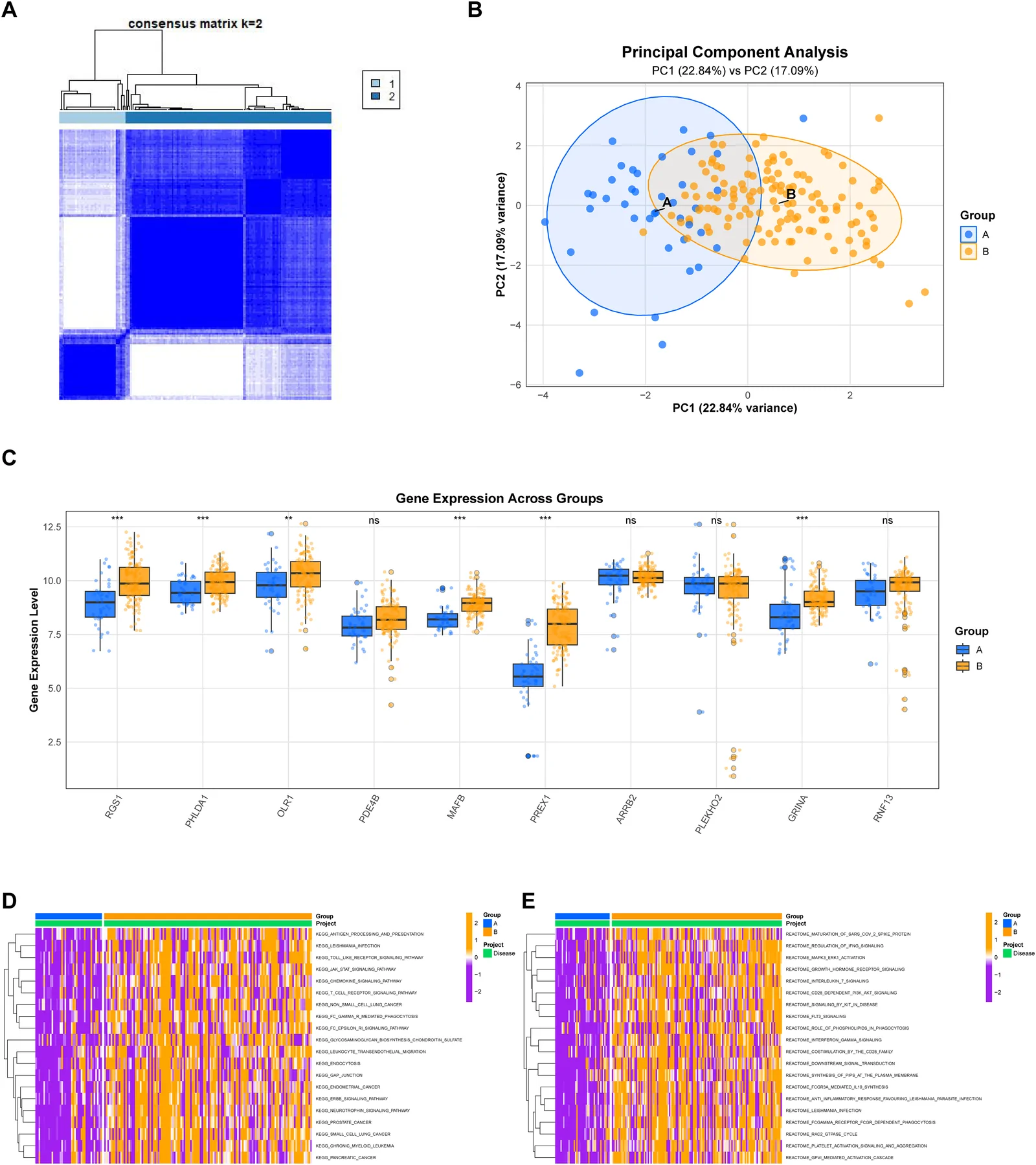
Transcriptomic clustering analysis of PAH based on candidate genes. **(A)** Consensus matrix heatmap showing the clustering results for k = 2, with clear separation between Group A and Group B **(B)** PCA plot illustrating the distribution of the two groups in the principal component space. **(C)** Box plots comparing the expression levels of the 10 candidate genes between the two groups. **(D, E)** Heatmaps showing the differences in KEGG **(D)** and Reactome **(E)** pathway activities between the two groups. Student’s t-test was used for comparison between groups. n = 257 expression profiles from 189 unique subjects. **P < 0.01, ***P < 0.001.

### 3.6 Whole-lung candidate gene expression validation in the MCT-induced PAH rat model

To experimentally validate the expression of the candidate genes identified from the integrative transcriptomic analysis, an MCT-induced PAH rat model was established. As shown in **Figure 14A**, RVSP was markedly elevated in MCT-treated rats compared with controls, confirming the development of PAH. Consistently, the RVHI was markedly increased in the MCT group (**Figure 14B**), indicating right ventricular hypertrophy secondary to pressure overload. Pronounced vascular wall thickening and luminal narrowing in pulmonary arterioles were observed in MCT-treated rats by histological examination of lung tissues (**Figure 14C**), which was further corroborated by quantitative morphometric analyses. Both WT% and WA% were significantly higher in the MCT group compared with controls (**Figure 14C**), confirming the presence of pulmonary vascular remodeling.

**Figure 14:**
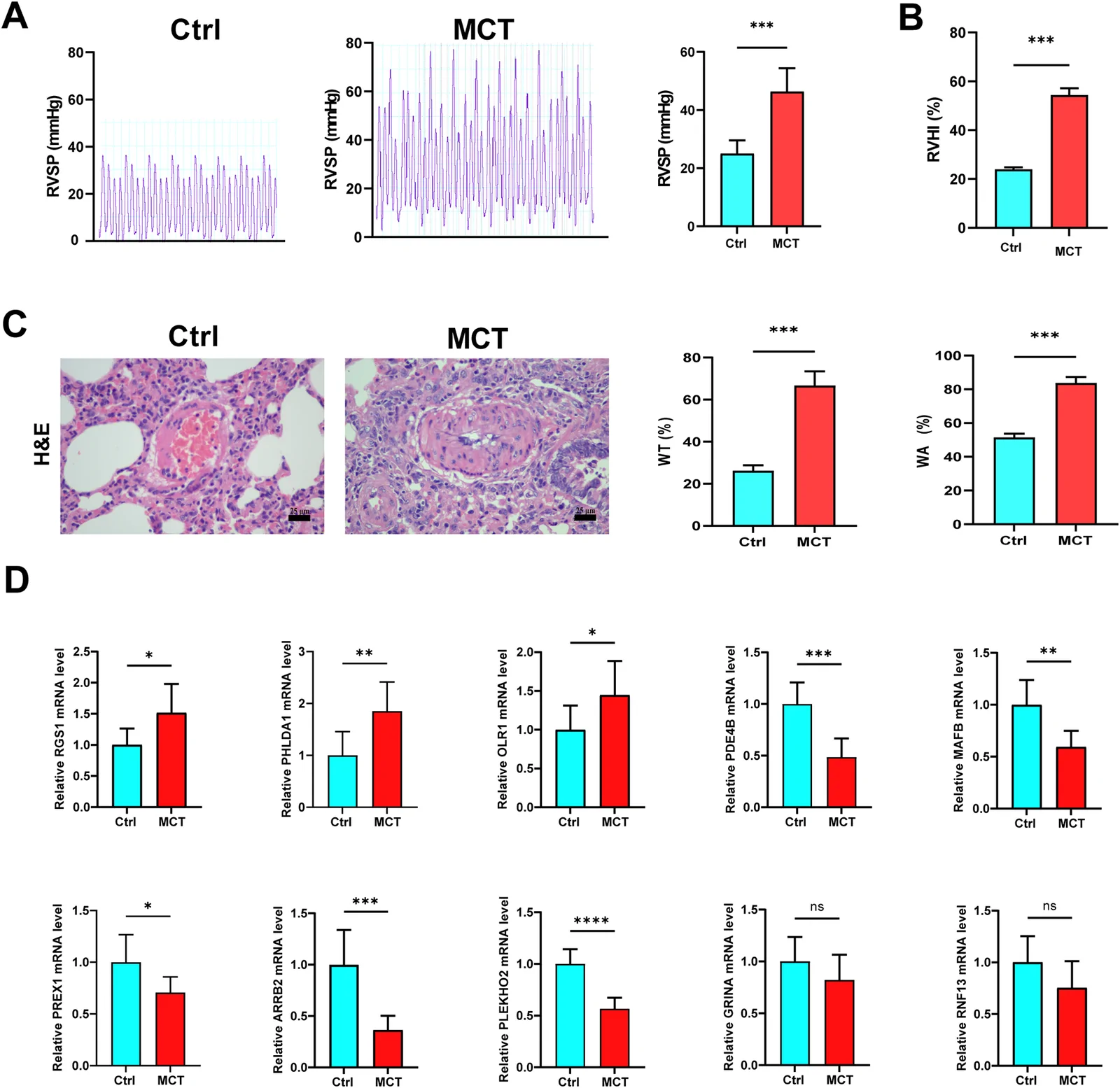
Whole-lung candidate gene expression validation in the MCT-induced PAH rat model. **(A)** Representative RVSP tracings (left) and quantitative comparison of RVSP between control and MCT groups (right). **(B)** Quantitative analysis of right ventricular hypertrophy index (RVHI), calculated as RV/(LV + IVS). **(C)** Representative photomicrographs of H&E-stained pulmonary arterioles (left; Scale bar: 25 µm) and quantitative analysis of vascular remodeling, including wall thickness percentage (WT%) and wall area percentage (WA%) (right). **(D)** RT-qPCR validation of the 10 candidate genes in lung tissues. Unpaired Student’s t-test was used for comparisons between control and MCT groups. Data are shown as mean ± SD (n = 8). ns, no significant, *P < 0.05, **P < 0.01, ***P < 0.001.

Finally, RT-qPCR analysis was performed to validate the expression of the 10 candidate genes in lung tissues (**Figure 14D**). Among these genes, *RGS1*, *PHLDA1*, and *OLR1* were significantly upregulated in MCT-treated rats compared with controls, whereas *MAFB*, *PREX1*, *ARRB2*, and *PLEKHO2* were significantly downregulated. *GRINA* and *RNF13* showed consistent downward trends without reaching statistical significance. Notably, *PDE4B* was the only gene exhibiting a discordant expression pattern, being significantly downregulated in MCT rats despite its upregulation in human PAH tissues. Overall, seven candidate genes showed significant direction-consistent changes, two showed direction-consistent nonsignificant trends, and one (*PDE4B*) was discordant, supporting tissue-level expression concordance.

## 4 Discussion

The mechanisms underlying cellular senescence in PAH remain poorly elucidated, especially with respect to cell-type-specific senescence heterogeneity and its functional roles. Herein, we systematically delineated a Mono.Macs-enriched senescence-associated gene landscape in PAH pulmonary vascular tissues by integrating single-cell transcriptomics with bulk transcriptomic datasets. Through machine learning integration, we prioritized a 10-gene candidate set with potential transcriptomic stratification value. Our findings suggest that a senescence-associated transcriptional signature in Mono.Macs may be linked to PAH and provide a prioritized candidate gene set for further investigation.

These single-cell observations are descriptive and should be interpreted as hypothesis-generating. In this study, we identified that DC, T.cells, and Mono.Macs exhibited the highest senescence-associated scores and were more frequently represented in the high senescence-associated score-defined group. Previous research has established that cellular senescence plays a pivotal role in pulmonary vascular remodeling and that targeting senescence-related pathways may offer therapeutic potential for PAH (40, 41). More recently, Safaie Qamsari et al. reported that endothelial cell senescence drives inflammation and vascular remodeling through the SASP in advanced PAH (10). Additionally, Kumar et al. have shown that interstitial macrophages are critical drivers of perivascular inflammation and vascular remodeling in hypoxic PH (42). Consequently, our findings support that cellular senescence in PAH involves not only vascular cells but also multiple immune cell populations, suggesting a broader cellular remodeling within the pulmonary vascular landscape. Further analysis showed that Mono.Macs with high senescence-associated scores exhibited lower CytoTRACE stemness scores, suggesting impaired functional plasticity. Senescence has also been associated with metabolic and functional alterations in macrophages, characterized by a shift toward glycolytic metabolism and reduced phagocytic capacity (13). Furthermore, senescent macrophages exhibit overexpression of SASP components (e.g., MCP-1, IL-6, TNF-α), thereby intensifying local inflammatory signals and establishing a “senescence-inflammation” positive feedback loop. Notably, while Mono.Macs exhibited a pronounced senescence signature at the single-cell level, bulk transcriptome analysis revealed a lower ssGSEA enrichment score for Mono.Macs in PAH lung tissues. This discrepancy may reflect a redistribution of senescence-associated Mono.Macs, although this interpretation remains speculative and would require spatial transcriptomics for validation. This interpretation aligns closely with the neighborhood enrichment identified through miloR analysis. Prior research has demonstrated that blood-borne monocytes are recruited to lung perivascular spaces, where they differentiate into inflammatory macrophages, a critical mechanism driving macrophage expansion in PH (12). Consequently, we hypothesize that senescence-associated Mono.Macs exhibit enrichment within specific transcriptomic neighborhoods in PAH, rather than a global numerical advantage. This hypothesis requires spatial validation.

CellChat analysis inferred communication patterns associated with the high senescence-associated score group, with an increase in both the number and strength of predicted interactions. These results suggest that senescence-associated changes are accompanied by altered intercellular communication patterns within the pulmonary vascular context. Ligand-receptor pairs such as CXCL1-ACKR1, CLEC2C-KLRB1, and MIF-CD74-CXCR4 were more prominent in the inferred communication of the high-score group, suggesting an intensification of chemokine-mediated immune recruitment and inflammatory signaling within a senescence-associated signaling context. ACKR1 regulates local chemokine gradients and leukocyte trafficking (43). Moreover, MIF, a pleiotropic inflammatory mediator, promotes pulmonary vascular remodeling and inflammatory activation through CD74-dependent signaling in PAH (44, 45). In addition, previous studies have shown that CXCL12-CXCR4 signaling contributes to pulmonary vascular remodeling in PAH, further supporting a role for CXCR4-related signaling in the senescence-associated signaling context (46). At the pathway level, signaling through MHC-II, COLLAGEN, and LAMININ exhibited the most significant increases, suggesting active processes of antigen presentation and extracellular matrix (ECM) remodeling within the senescence-associated signaling context. Interstitial macrophages with high MHC-II expression have been implicated in pulmonary arterial medial fibrosis and vascular remodeling in PAH (47), while increased COLLAGEN and LAMININ signaling may indicate dysregulated basement membrane remodeling that contributes to endothelial dysfunction and pulmonary vascular remodeling in PAH (48). Immune recruitment, immune activation, and ECM remodeling were coordinately activated in the high senescence-associated score group. This network may disrupt pulmonary vascular homeostasis and potentially participate in pathological vascular remodeling in PAH (10). These findings describe inferred communication patterns associated with the senescence-associated score and do not establish causal enhancement of communication by senescence. Composition and cell-number sensitivity analyses were not performed for CellChat inference; therefore, these inferred communication patterns should be interpreted as descriptive and exploratory, and may partly reflect differences in cell composition or cell numbers between groups.

To elucidate the key molecules associated with Mono.Macs senescence in PAH, we combined correlation filtering with differential expression analysis to identify senescence-related genes enriched in Mono.Macs. This yielded 405 candidate genes with Mono.Macs-enriched expression patterns in the single-cell dataset, 34 of which were further identified as significantly dysregulated in the discovery-stage bulk transcriptomic datasets. Together, these results suggest that Mono.Macs may be important contributors to the senescence-associated transcriptional landscape in PAH. Integrative analysis of single-cell and bulk transcriptomic data has been widely applied in PAH and other complex diseases, enhancing the robustness and biological plausibility of candidate gene identification, and supporting the importance of cell-type-specific analyses in understanding disease mechanisms (16, 49). We then employed machine learning techniques, such as LASSO, Random Forest, and XGBoost, to refine the candidate genes into a 10-gene candidate set. The machine-learning component of this study was designed to prioritize candidate genes through cross-method agreement rather than to develop a clinical prediction model. Cross-method concordance was used to improve the robustness of candidate gene selection, consistent with previous transcriptomic studies in PAH biomarker discovery (50, 51). Agreement among the three algorithms does not constitute independent validation but serves to prioritize candidates for subsequent evaluation.

The robustness of the prioritized candidates was further supported by donor-level consistency (all 10 genes positively associated with the UCell score across six donors) and by a sensitivity analysis restricted to 189 unique subjects, in which all 10 genes retained their discovery-stage direction and significance after within-gene-set BH correction. Independent expression analyses reproduced the expected directions of *RGS1*, *PHLDA1*, *MAFB*, and *RNF13* in one or more external cohorts, providing additional support for their prioritization. After within-cohort multiple-testing correction, seven gene-cohort combinations retained directional consistency with the discovery-stage findings, including *RGS1*, *PHLDA1*, *MAFB*, and *RNF13* in lung tissue cohorts, and MAFB in a PBMC cohort, whereas the remaining combinations were either nonsignificant or discordant. In the discovery-stage data, *RGS1* showed the highest discriminatory capacity (AUC = 0.818), although this should be interpreted as a discovery-stage observation rather than validated clinical utility. These genes should be interpreted as biologically informed candidates for subsequent mechanistic investigation. In addition, these candidate genes may be linked to inflammatory activation, immune remodeling, and metabolic alterations associated with Mono.Macs senescence.

In addition to the computational validation using public bulk datasets, we further evaluated the expression of the 10 candidate genes in an *in vivo* experimental model. In MCT-induced PAH rats, the PAH phenotype was successfully recapitulated, as evidenced by elevated RVSP, increased RVHI, and pulmonary vascular remodeling, confirming the suitability of this model for molecular validation. RT-qPCR analysis of lung tissues revealed seven significant direction-consistent changes, two direction-consistent nonsignificant trends, and one discordant gene (*PDE4B*), supporting tissue-level expression concordance. Specifically, *RGS1*, *PHLDA1*, and *OLR1* were significantly upregulated, whereas *MAFB*, *PREX1*, *ARRB2*, and *PLEKHO2* were significantly downregulated in MCT rats. *GRINA* and *RNF13* showed consistent downward trends without reaching statistical significance, which may reflect the dilution of cell-type-specific signals in whole-lung homogenates or limited sample size. These results support tissue-level expression concordance of candidate genes across species in the context of PAH pathology. It is important to note that these whole-lung RT-qPCR results support expression concordance at the tissue level, but do not localize the observed changes to Mono.Macs or establish a causal role in vascular remodeling.

Beyond expression validation, we further explored the potential functional relevance of these candidate genes in Mono.Macs senescence-associated signature by examining several representative candidates. *RGS1*, a regulator of G-protein signaling, contributes to the chemotaxis and retention of myeloid cells. Previous studies have demonstrated that *RGS1* inhibits macrophage migration by attenuating chemokine receptor signaling, thereby promoting sustained inflammatory cell retention at sites of injury (52). This mechanism is consistent with our observation of an enrichment of senescence-associated Mono.Macs in specific transcriptional neighborhoods, although this remains speculative without spatial validation. ARRB2 (β-arrestin-2) is highly expressed in macrophages and regulates macrophage survival while also contributing to TLR-induced complement C1q expression (53). In our study, ARRB2 was downregulated in Mono.Macs with high senescence-associated scores, which might be linked to the accumulation of Mono.Macs with senescence-associated features by enhancing cell survival and impairing immune effector function. Interestingly, the role of ARRB2 appears to be cell type dependent. BMPR2 deficiency has been reported to promote a hyperproliferative and hypocontractile PASMC phenotype through ARRB2-associated signaling pathways (54). These observations indicate that ARRB2 regulates inflammatory responses and cellular retention in immune cells, while also contributing to vascular remodeling in structural cells.

*MAFB* is a key transcription factor involved in macrophage differentiation and polarization. *MAFB* deficiency impairs macrophage maturation and disrupts immune function (55). In this study, lower *MAFB* expression was associated with the senescence-associated transcriptional signature, suggesting a possible association with impaired maturation and reduced functional plasticity in Mono.Macs. *RNF13*, an E3 ubiquitin ligase, enhances endosomal Toll-like receptor (TLR)-mediated inflammatory signaling in macrophages by promoting K48-linked polyubiquitination and subsequent degradation of LAMP-1, thereby inhibiting lysosomal maturation (56). This mechanism has mainly been described under classical inflammatory conditions in macrophages. In Mono.Macs with high senescence-associated scores, lower *RNF13* expression was associated with enrichment of pathways related to cell cycle regulation, homologous recombination repair, and cellular senescence. *RNF13* likely contributes not only to inflammatory regulation in PAH but also to cellular homeostasis and cellular senescence-associated transcriptional programs. *OLR1* (LOX-1), an innate immune scavenger receptor, is involved in oxidized low-density lipoprotein-induced vascular endothelial damage and inflammatory processes, and its upregulation has been documented in various vascular pathologies (57). In PAH, *OLR1* has been associated with hypoxia-induced phenotypic switching of PASMCs and subsequent vascular remodeling (58). In our study, *OLR1* expression was elevated in Mono.Macs with high senescence-associated scores, suggesting a role in vascular inflammation and remodeling of the local cellular milieu. Furthermore, *PDE4B* is a key regulator of TLR signaling and tumor necrosis factor-alpha (TNF-α) production (59). Its increased expression in Mono.Macs with high senescence-associated scores indicates that *PDE4B* may contribute to persistent immune activation in PAH by enhancing TLR-mediated inflammatory responses. Of note, *PDE4B* was upregulated in human PAH but downregulated in MCT rats. This discordance may reflect differential immune cell infiltration: *PDE4B* is highly expressed in granulocytes and DCs, which predominate in the acute MCT model, whereas chronic human PAH is driven by monocyte/macrophage accumulation. These findings highlight the importance of cell-type-specific validation in future studies.

The 10 candidate genes exhibited coordinated yet heterogeneous expression patterns in Mono.Macs with high senescence-associated scores, suggesting potential involvement in the altered immune landscape observed in PAH. It should be noted that the biological functions of these candidate genes remain to be investigated through *in vitro* knockdown/overexpression experiments and cell co-culture systems. At present, only *ARRB2* has available drug perturbation data, whereas the remaining genes lack clearly defined pharmacological targeting information, which reflects the limitations of current databases. We also acknowledge that the senescence scoring method is gene-set dependent and that the median threshold and subsequent gene-score correlations may carry some degree of mathematical coupling, although the multi-layer filtering strategy partially mitigates this dependency. The robustness of the senescence score was supported by an independent UCell rank-based scoring approach, which showed high correlation with the original AddModuleScore (Spearman rho = 0.804) and consistent positive associations with all 10 candidate genes across donors. In addition, none of the 10 candidate genes were members of the 321-gene senescence set, indicating that the gene-score associations were not driven by direct mathematical coupling at the gene level. Limited pathway-level overlap was observed (e.g., inflammatory response and IL6-JAK-STAT3 signaling); however, after excluding overlapping genes, the associations between the senescence-associated score and these pathways remained directionally consistent across all six donors. These genes may represent candidate targets for future therapeutic investigation, and further experimental studies, including CRISPR-based functional screening and pharmacological intervention, are needed to evaluate their therapeutic relevance in PAH.

Beyond functional characterization, we further explored the potential transcriptomic stratification value of these candidate genes. Consensus clustering based on the 10 candidate genes classified patients with PAH in the bulk transcriptomic datasets into two exploratory transcriptomic groups: Group A, characterized by relatively low expression of genes such as *RGS1* and *PHLDA1*, and Group B, characterized by higher expression of these genes. KEGG and Reactome pathway analyses indicated that, compared with Group A, Group B displayed higher enrichment in multiple immune-inflammatory pathways, including FcγR-mediated phagocytosis, JAK-STAT signaling, T-cell receptor signaling, and interferon-γ signaling. Based on these features, Group B was tentatively referred to as an “inflammation-enriched” group, whereas Group A was referred to as an “inflammation-low” group. These findings suggest potential transcriptomic heterogeneity among patients with PAH. Several preclinical studies have investigated anti-inflammatory interventions in PAH models, including the JAK inhibitor ruxolitinib and TNF-α antagonists (60–62). While these studies suggest that inflammatory pathways may be pharmacologically targetable in PAH, the relevance of these findings to the transcriptomic groups identified in our study remains unknown and would require prospective investigation. The inflammation-low group showed relatively lower enrichment of inflammatory pathways compared with the inflammation-enriched group. Whether this difference reflects distinct pathobiological mechanisms or has therapeutic implications remains to be determined. Metabolic reprogramming is increasingly recognized as a central pathological feature of PAH and a potential therapeutic target (63). Meanwhile, senolytic therapy has emerged as a potential therapeutic strategy in PAH (64). The senescence-associated exploratory transcriptomic groups identified in this study may provide a hypothesis-generating basis for future investigations into senescence-targeted approaches. Of note, previous studies have also identified distinct exploratory transcriptomic groups of idiopathic pulmonary arterial hypertension (IPAH) with different prognostic and transcriptomic features based on unsupervised clustering analyses (65). These findings further support the presence of transcriptomic heterogeneity in PAH. However, the associations between these exploratory transcriptomic groups and clinical characteristics, including hemodynamic parameters and survival outcomes, remain to be further investigated.

Several limitations of this study should be acknowledged. First, the single-cell RNA-seq dataset includes only three PAH patients and three control donors, which limits donor-level statistical inference and prevents robust pseudobulk or mixed-effect modeling. Although the external bulk cohorts and the MCT rat model provided orthogonal support for the prioritized candidates, the small donor number in the single-cell analysis underscores the need for future studies with larger single-cell cohorts and donor-level analytical approaches to confirm the reproducibility of our findings. Second, the functional relevance of the prioritized candidate genes remains to be established; future cell-type-specific knockdown/overexpression and co-culture experiments are needed to establish their mechanistic contribution to PAH.

In summary, this study systematically characterized a Mono.Macs-centered senescence-associated gene landscape in PAH pulmonary vascular tissues and prioritized a 10-gene candidate set with potential transcriptomic stratification value. Tissue-level expression concordance of these candidates was supported by whole-lung RT-qPCR validation in an MCT-induced PAH rat model. Mono.Macs with a senescence-associated transcriptional signature may be linked to vascular remodeling through altered inferred intercellular communication, whereas candidate gene-based exploratory transcriptomic grouping may provide a hypothesis-generating basis for future investigations. Further studies are needed to validate the biological relevance of these candidates, including cell-type-specific and functional investigations, as well as prospective evaluation of their transcriptomic stratification value in independent PAH cohorts.

## Data Availability

The public datasets analyzed in this study are available in GEO under accession numbers GSE210248, GSE113439, GSE117261, GSE254617, GSE15197, GSE33463, GSE48149, GSE53408, GSE22356, GSE131793, and GSE38267. The complete analytical code has been organized as Supplementary Code. Sample annotations, probe-to-gene mappings, duplicate-subject audit tables, and targeted gene-expression results are available from the corresponding author upon reasonable request.
